# Correction: Hyperspectral technology and machine learning models to estimate the fruit quality parameters of mango and strawberry crops

**DOI:** 10.1371/journal.pone.0345011

**Published:** 2026-03-12

**Authors:** Salah Elsayed, Hoda Galal, Mohamed S. Abd El-baki, Mohamed Maher Ibrahim, Ahmed Elbeltagi, Ali Salem, Abdallah Elshawadfy Elwakeel, Osama Elsherbiny, Nadia G. Abd El-Fattah

The second and fourth authors’ names are spelled incorrectly. The correct names are: Hoda Galal and Mohamed Maher Ibrahim. The correct citation is: Elsayed S, Galal H, Abd El-baki MS, Ibrahim MM, Elbeltagi A, Salem A, et al. (2025) Hyperspectral technology and machine learning models to estimate the fruit quality parameters of mango and strawberry crops. PLoS ONE 20(2): e0313397. https://doi.org/10.1371/journal.pone.0313397
