# Supplementary material for: Hyperspectral technology and machine learning models to estimate the fruit quality parameters of mango and strawberry crops
Source: PLoS One. 2025 Feb 11;20(2):e0313397. doi: 10.1371/journal.pone.0313397 (PMC11813159; doi:10.1371/journal.pone.0313397)
Supplement: Attachment — Submitted filename: PONE-D-24-31212_reviewer.pdf [file pone.0313397.s001.pdf]

PLOS ONE

Hyperspectral Technology and Machine Learning Models to Estimate the Fruit Quality Parameters of Mango and Strawberry Crops

--Manuscript Draft--

Manuscript Number:	PONE-D-24-31212
Article Type:	Research Article
Full Title:	Hyperspectral Technology and Machine Learning Models to Estimate the Fruit Quality Parameters of Mango and Strawberry Crops
Short Title:	Hyperspectral Technology and Machine Learning Models to Estimate the Fruit Quality
Corresponding Author:	Ahmed Elbeltagi<br>Mansoura University Faculty of Agriculture<br>Aga, EGYPT
Keywords:	artificial neural networks; decision tree; random forest; Mango, strawberry, spectral indices.
Abstract:	Using chemical laboratory procedures to estimate the fruit quality parameters (biochemical parameters) of mango "Succarri" and strawberry "Florida" as indicators of ripening degrees in a large area presents challenges such as low throughput, labor intensity, time consumption, and the need for multiple samples. So, using spectral reflectance-based proximal remote sensing to quickly and accurately measure biochemical parameters in different fruits is important to find the best time to harvest, make food ripen faster, and the processing of food easier. This has significant economic and ecological advantages. The objective of this study was to evaluate the biochemical parameters of mango and strawberry fruits at various ripening stages. This was done by utilizing a combination of established and newly developed spectral reflectance indices (SRIs) in conjunction with machine learning (ML) models, including artificial neural networks (ANN), random forests (RF), and decision trees (DT). For mango fruit, the parameters estimated were chlorophyll content, total soluble solids (TSS), and firmness, whereas for strawberry fruit, the parameters were L*, b*, TSS, and firmness. These results revealed significant differences in SRI values across various ripening stages, indicating variances in the fruit’s biochemical parameters. The newly developed SRIs showed superior efficacy in evaluating these parameters. The integration of SRIs with diverse ML models proved to be a successful strategy for precisely estimating biochemical parameters. For mango's biochemical parameter prediction, the ANN models demonstrated R2 values ranging from 0.92 to 1.00 and from 0.93 to 0.98 for training and testing, respectively. On the other hand, the RF models exhibited R2 values ranging from 0.98 to 1.00 and from 0.93 to 0.99 during training and testing, respectively. The DT models showed high performance, with R2 values ranging from 0.95 to 1.00 and from 0.88 to 0.99 for the training and testing phases. For strawberry's biochemical parameter prediction, the ANN models achieved R2 values between 0.75 and 0.91 and between 0.58 and 0.91 during training and testing phases, respectively. On the other hand, RF models showed R2 values between 0.85 and 0.91 during training and between 0.74 and 0.86 during testing. The DT models demonstrated excellent results, with R2 values ranging from 0.75 to 0.91 for the training set and 0.74 to 0.81 for the testing set. It can be concluded that combining SRIs with ML models, such as ANN, RF, and DT, can accurately predict the biochemical properties of mango and strawberry fruits.
Order of Authors:	Salah Elsayed
	Hoda Gala
	Mohamed S. Abd El-baki
	Mohamed Maher
	Ahmed Elbeltagi
	Ali Salem
	Abdallah Elshawadfy Elwakeel

Powered by Editorial Manager® and ProduXion Manager® from Aries Systems Corporation

	Osama Elsherbiny
	Nadia G. Abd El-Fattah
**Additional Information:**	
**Question**	**Response**
**Financial Disclosure**<br><br>Enter a financial disclosure statement that describes the sources of funding for the work included in this submission. Review the submission guidelines for detailed requirements. View published research articles from *PLOS ONE* for specific examples.<br><br>This statement is required for submission and **will appear in the published article** if the submission is accepted. Please make sure it is accurate.<br><br>**Funded studies**<br>Enter a statement with the following details:<br>• Initials of the authors who received each award<br>• Grant numbers awarded to each author<br>• The full name of each funder<br>• URL of each funder website<br>• Did the sponsors or funders play any role in the study design, data collection and analysis, decision to publish, or preparation of the manuscript?<br><br>Did you receive funding for this work?	The author(s) received no specific funding for this work.
**Competing Interests**<br><br>Use the instructions below to enter a competing interest statement for this submission. On behalf of all authors, disclose any competing interests that could be perceived to bias this work—acknowledging all financial support and any other relevant financial or non-financial competing interests.<br><br>This statement is required for submission and **will appear in the published article** if	The authors have declared that no competing interests exist

the submission is accepted. Please make sure it is accurate and that any funding sources listed in your Funding Information later in the submission form are also declared in your Financial Disclosure statement.<br><br>View published research articles from *PLOS ONE* for specific examples.<br><br>**NO authors have competing interests**<br><br>Enter: *The authors have declared that no competing interests exist.*<br><br>**Authors with competing interests**<br><br>Enter competing interest details beginning with this statement:<br><br>*I have read the journal's policy and the authors of this manuscript have the following competing interests: [insert competing interests here]*<br><br>* typeset	
**Ethics Statement**<br><br>Enter an ethics statement for this submission. This statement is required if the study involved:<br><br>• Human participants<br>• Human specimens or tissue<br>• Vertebrate animals or cephalopods<br>• Vertebrate embryos or tissues<br>• Field research<br><br>Write "N/A" if the submission does not require an ethics statement.<br><br>General guidance is provided below. Consult the submission guidelines for detailed instructions. **Make sure that all information entered here is included in the Methods section of the manuscript.**	N/A

**Format for specific study types**<br><br>**Human Subject Research (involving human participants and/or tissue)**<br>• Give the name of the institutional review board or ethics committee that approved the study<br>• Include the approval number and/or a statement indicating approval of this research<br>• Indicate the form of consent obtained (written/oral) or the reason that consent was not obtained (e.g. the data were analyzed anonymously)<br><br>**Animal Research (involving vertebrate animals, embryos or tissues)**<br>• Provide the name of the Institutional Animal Care and Use Committee (IACUC) or other relevant ethics board that reviewed the study protocol, and indicate whether they approved this research or granted a formal waiver of ethical approval<br>• Include an approval number if one was obtained<br>• If the study involved *non-human primates*, add *additional details* about animal welfare and steps taken to ameliorate suffering<br>• If anesthesia, euthanasia, or any kind of animal sacrifice is part of the study, include briefly which substances and/or methods were applied<br><br>**Field Research**<br><br>Include the following details if this study involves the collection of plant, animal, or other materials from a natural setting:<br>• Field permit number<br>• Name of the institution or relevant body that granted permission	
**Data Availability**<br><br>Authors are required to make all data underlying the findings described fully available, without restriction, and from the time of publication. PLOS allows rare exceptions to address legal and ethical concerns. See the PLOS Data Policy and FAQ for detailed information.	Yes - all data are fully available without restriction

Powered by Editorial Manager® and ProduXion Manager® from Aries Systems Corporation

A Data Availability Statement describing where the data can be found is required at submission. Your answers to this question constitute the Data Availability Statement and **will be published in the article**, if accepted.<br><br>**Important:** Stating 'data available on request from the author' is not sufficient. If your data are only available upon request, select 'No' for the first question and explain your exceptional situation in the text box.<br><br>Do the authors confirm that all data underlying the findings described in their manuscript are fully available without restriction?	
**Describe where the data may be found in full sentences. If you are copying our sample text, replace any instances of XXX with the appropriate details.**<br><br>• If the data are **held or will be held in a public repository**, include URLs, accession numbers or DOIs. If this information will only be available after acceptance, indicate this by ticking the box below. For example: *All XXX files are available from the XXX database (accession number(s) XXX, XXX.*).<br>• If the data are all contained **within the manuscript and/or Supporting Information files**, enter the following: *All relevant data are within the manuscript and its Supporting Information files.*<br>• If neither of these applies but you are able to provide **details of access elsewhere**, with or without limitations, please do so. For example:<br><br>*Data cannot be shared publicly because of [XXX]. Data are available from the XXX Institutional Data Access / Ethics Committee (contact via XXX) for researchers who meet the criteria for access to confidential data.*<br><br>*The data underlying the results presented in the study are available from (include the name of the third party*	Data will be available upon request

and contact information or URL).<br>• This text is appropriate if the data are owned by a third party and authors do not have permission to share the data.<br><br>* typeset	
Additional data availability information:	

Manuscript Click here to access/download;Manuscript;Manuscript 1.docx

Hyperspectral Technology and Machine Learning Models to Estimate the Fruit Quality Parameters of Mango and Strawberry Crops

Salah Elsayed [1], Hoda Gala [2,3], Mohamed S. Abd El-baki [4], Mohamed Maher [4], Ahmed Elbeltagi[4,*], Ali Salem [5,6,*], Abdallah Elshawadfy Elwakeel [7], Osama Elsherbiny [4], Nadia G. Abd El-Fattah [4]

[1] Agriculture Engineering, Evaluation of Natural Resources Department, Environmental Studies and Research Institute, University of Sadat City, Menoufia 32897, Egypt; salah.emam@esri.usc.edu.eg

[2] New Era and Development in Civil Engineering Research Group, Scientific Research Center, Al-Ayen University, Nasiriyah, Thi-Qar 64001, Iraq

[3] Pomology, Evaluation of Natural Resources Department, Environmental Studies and Research Institute, University of Sadat City, Menoufia 32897, Egypt; hoda.galal@esri.usc.edu.eg

[4] Agricultural Engineering Department, Faculty of Agriculture, Mansoura University, Mansoura 35516, Egypt; mohamedsalah@mans.edu.eg; mohamed_maher@mans.edu.eg; ahmedelbeltagy81@mans.edu.eg; Nadia_gamal91@mans.edu.eg; _osama_algazeery@mans.edu.eg

[5] Structural Diagnostics and Analysis Research Group, Faculty of Engineering and Information Technology, University of Pecs, Hungary

[6] Civil Engineering Department, Faculty of Engineering, Minia University, Egypt, salem.ali@mik.pte.hu

[7] Agricultural Engineering Department, Faculty of Agriculture and Natural Resources, Aswan University, Aswan 81528, Egypt; abdallah_elshawadfy@agr.aswu.edu.eg

Correspondence: salem.ali@mik.pte.hu (A.S.); ahmedelbeltagy81@mans.edu.eg (A.E.)

Abstract: Using chemical laboratory procedures to estimate the fruit quality parameters (biochemical parameters) of mango "Succarri" and strawberry "Florida" as indicators of ripening degrees in a large area presents challenges such as low throughput, labor intensity, time consumption, and the need for multiple samples. So, using spectral reflectance-based proximal remote sensing to quickly and accurately measure biochemical parameters in different fruits is important to find the best time to harvest, make food ripen faster, and the processing of food easier. This has significant economic and ecological advantages. The objective of this study was to evaluate the biochemical parameters of mango and strawberry fruits at various ripening stages. This was done by utilizing a combination of established and newly developed spectral reflectance indices (SRIs) in conjunction with machine learning (ML) models, including artificial neural networks (ANN), random forests (RF), and decision trees (DT). For mango fruit, the parameters estimated were chlorophyll content, total soluble solids (TSS), and firmness, whereas for strawberry fruit, the parameters were L*, b*, TSS, and firmness. These results revealed significant differences in SRI values across various ripening stages, indicating variances in the fruit's biochemical parameters. The newly developed SRIs showed superior efficacy in evaluating these parameters. The integration of SRIs with diverse ML models proved to be a successful strategy for precisely estimating biochemical parameters. For mango's biochemical parameter prediction, the ANN models demonstrated R^2 values ranging from 0.92 to 1.00 and from 0.93 to 0.98 for training and testing, respectively. On the other hand, the RF models exhibited R^2 values ranging from 0.98 to 1.00 and from 0.93 to 0.99 during training and testing, respectively. The DT models showed high performance, with R^2 values ranging from 0.95 to 1.00 and from 0.88 to 0.99 for the training and testing phases. For strawberry's biochemical parameter prediction, the ANN models achieved R^2 values

between 0.75 and 0.91 and between 0.58 and 0.91 during training and testing phases, respectively. On the other hand, RF models showed R^2 values between 0.85 and 0.91 during training and between 0.74 and 0.86 during testing. The DT models demonstrated excellent results, with R^2 values ranging from 0.75 to 0.91 for the training set and 0.74 to 0.81 for the testing set. It can be concluded that combining SRIs with ML models, such as ANN, RF, and DT, can accurately predict the biochemical properties of mango and strawberry fruits.

Keywords: artificial neural networks; decision tree; random forest; Mango, strawberry, spectral indices.

1. Introduction

Mango and strawberry are two of the most preferred fruit crops worldwide. Population consumes fresh or utilized in the production of jam, juice, and marmalade, as well as in the manufacturing of other food products. They could be consumed in local markets or transferred to foreign countries. Therefore, it is necessary to harvest fruits at a suitable ripening stage for each case. Since during the ripening process, several physiological and chemical changes take place, such as alterations in fruit hardness, total soluble solids, and titratable acidity, as well as variations in fruit color and aroma. Fruit firmness and titratable acidity exhibit high levels in immature and mature green fruits and decrease significantly during the ripening stage. On the other hand, total soluble solids increased as ripening progressed [1]. Fruits are categorized into climacteric and non-climacteric categories based on their respiration patterns, which correlate with ripening. Mangoes (*Mangifera indica* L.) are a prototypical climacteric fruit. During the process of ripening, there is a distinct climacteric peak characterized by a sudden rise in the intensity of respiration, connected to the rapid production of ethylene [2]. Mangoes can be harvested in a mature green stage and then continue to ripen after being harvested [1]. On the other side, strawberries) *Fragaria ananassa* Duch.) are considered non-climacteric fruits, meaning they have no change in respiration, and have very low levels of ethylene production. Therefore, they should be harvested after ripening [2]. Van de Poel et al. [3] discovered that strawberries can ripen after being detached at the green stage, however, this process significantly impacts fruit quality. Strawberries that were separated from the plant did not grow to the same size, had low levels of malic acid, glucose, fructose, and sucrose, and had fewer volatile compounds. As a result, they had a less intense aroma and an undesirable taste compared to strawberries that ripened on the vine.

Traditionally, mango and strawberry fruits were typically assessed by analyzing their chemical properties with specialized equipment like refractometers and colorimeters [4,5]. Accurately assessing fruit ripeness using traditional methods is challenging due to the requirement for extensive, representative samples and skilled operators. This makes these methods destructive, uneconomical, and impractical for large-scale use. As a result, research has shifted towards replacing traditional methods with nondestructive, rapid, low-cost, and real-time decision-making alternative methods [6–8].

In recent years, proximal remote sensing techniques have become a promising alternative to traditional methods for detecting crucial traits closely associated with mango and strawberry fruit ripeness and determining the best time for harvest [9,10]. These techniques have the potential to rapidly and non-invasively gather various characteristics related to mango and strawberry fruit ripeness for a large number of samples on a large scale [11,12]. Among these techniques is the use of portable VIS/NIR or NIR spectroscopy, equipped with multiple sensors that can detect variations in the optical characteristics of the fruit's surface at different wavelengths. Therefore, we

could use the spectra emitted by the fruit's surface for the direct or indirect assessment of various traits associated with mango and strawberry ripeness.

As mango and strawberry fruits ripen, their metabolic status undergoes various changes. These changes include alterations in cell wall structure, a decrease in chlorophyll content, and firmness, as well as a gradual increase in TSS. These changes result in significant changes in the spectral reflectance of the fruit across the electromagnetic spectrum [13–15]. People commonly observe changes in the spectral reflectance of ripening grapes in the visible spectrum (VIS, 400–700 nm), as well as the red edge (650–720 nm) and near-infrared (NIR, 700–1200 nm) regions of the spectrum [16]. This suggests that a spectroscopy device that covers only the VIS spectrum, and/or the VIS and NIR spectrums could be useful for detecting changes in the characteristics of fruits during their ripeness. For example, Fatchurrahman et al. [17] found that the spectroscopy of VIS/NIR (400–1000 nm) and NIR (900–1700 nm) was effective in assessing the content of TSS and phenols in goji berries (*Lycium barbarum* L.) at four different maturity stages.

Generally, the most effective wavelengths within the VIS and NIR spectrum are commonly used in specific equations to create specific spectral reflectance indices (SRIs) such as the anthocyanin index (NAI), normalized chlorophyll index (NCI), normalized difference vegetation index (NDVI), pigment sensitive ripening monitoring index (PRMI), and greenness index (GI) [18–20]. These SRIs are commonly used to monitor changes in biochemical and biophysical attributes during fruit ripeness [21–25]. For example, Elsayed et al. [25] found that the normalized spectral indices HPS 760 - 730, 760- 720 and 686-620 showed the highest coefficients of determination for the SPAD value, chlorophyll a, chlorophyll b and chlorophyll t of mango fruits of the Zabdia cultivar. Additionally, the two-band $SRI_{450,640}$, calculated from the blue and red regions in the VIS spectrum, showed the highest R^2 values (0.95 and 0.90) for TSS and firmness, respectively, in ripening banana fruits.

The assessment of fruit quality in crops using SRIs often yields inconsistent outcomes in diverse geographical and environmental conditions. Therefore, there is an ongoing need for refining SRIs to enhance their effectiveness as a rapid and straightforward approach for accurately estimating fruit quality parameters. It is of utmost importance to ascertain the optimal algorithmic formulations for the computation of diverse fruit quality attributes, thereby enhancing the efficacy of remotely acquired data in the evaluation of fruit quality [26,27]. Typically, prior studies have primarily concentrated their efforts on utilizing published SRIs for the assessment of various fruit quality attributes. As far as the authors are aware, only a limited number of inquiries have investigated the simultaneous application of distinct techniques for fruit property assessment. The distinctive advantage of the present investigation lies in the methodology employed for selecting the most suitable SRIs for the evaluation of fruit quality parameters. In this regard, the utilization of correlogram maps stands out as a noteworthy approach, enhancing the study's capability to identify and employ the most effective SRIs in assessing fruit quality attributes.

Although SRIs offer a straightforward approach to the estimation of biochemical parameters, with the potential to enable the development of a portable and lightweight instrument for the rapid and cost-effective assessment and management of biochemical parameters on a significant scale, it is important to note that each SRI is constrained by a finite set of band combinations. The challenge lies in formulating robust SRIs for the assessment of fruit quality attributes amidst diverse and potentially perplexing conditions. These conditions encompass substantial variations in the dimensions of fruit components and their consequential impact on the saturation level of the quality parameters under scrutiny. As a subset of artificial intelligence (AI), machine learning (ML) has grown quickly in this environment. A huge amount of spectrum data can be used by

machine learning (ML) to extract important information for accurate classification and self-prediction [28].

Although multiple linear regression is a widely used technique, its ability to reliably forecast the intended output is limited by the non-linearity of its inputs. Machine learning (ML) models have experienced a surge in popularity in recent years and are now extensively utilized, particularly in domains like smart agriculture and the food business. Scala et al. [29] and Torkashvand et al. [30] found that ML models are effective in contributing to increasing the productivity and quality of fruits. ML models such as artificial neural networks (ANN), random forest (RF), and decision tree (DT) are known for their effectiveness in pattern recognition, prediction and classification of the quality of different fruits [31,32]. These approaches are highly proficient at acquiring and representing intricate, frequently non-linear connections between input and output signals, relying on given patterns [33,34]. it has the ability to learn from example datasets in an iterative manner, even without any prior information about the relationships between the process variables. Prior research has established the dependability and usefulness of these models in forecasting fruit attributes [27,30,35–37]. Based on these factors, our study seeks to employ artificial neural networks, random forest, and decision tree algorithms to analyze and define the quality parameters of strawberry and mango fruits.

In the context of this research study, the overarching objective was to assess the effectiveness of SRIs as non-destructive techniques for estimating the characteristics of mango and strawberry fruits, as well as for detecting the quality parameters of these fruits at various stages of maturity. To achieve this, the study set out to accomplish the following specific goals: (i) Quantify the quality parameters of mango and strawberry fruits at different stages of ripening; (ii) Evaluate the suitability of conventional and newly developed SRIs for quantifying the quality parameters of mango and strawberry fruits; and (iv) Assess the performance of ANN, DT and RF models, which are based on SRIs, in predicting the quality parameters of mango and strawberry fruits.

2. Materials and Methods

2.1. Samples Collection and Analytical Procedures

Mango "Succarri" and strawberry "Florida" fruits were collected from a private orchard placed at Cairo-Alexandria Desert Road. Seventy five samples of mango and forty five plates of strawberry fruits in different ripening stages, from mature green to fully ripening, were selected. The collected fruits were free from diseases, insects, or mechanical damage. Fruit samples were transferred directly to the lab to estimate the following parameters. Samples from each type under three different ripening degrees were selected to achieve the physical and chemical analysis.

The chlorophyll content or SPAD of mango was measured by using a portable chlorophyll meter, SPAD-502 (Konica-Minolta, Osaka, Japan). The firmness of each fruit for mango and strawberry was measured on different sides using a digital fruit hardness tester (IC-FR5120, China) with a 6 mm probe, and the result was recorded in Newton. Each fruit of mango and strawberry was squeezed separately to measure TSS. TSS was measured with a refractometer (Milwaukee, model MA871, Italy).

Peel color was measured using a precision colorimeter (Sucolor SC-10, China). Measurements were taken at three locations chosen at random for each fruit of strawberry, providing color space parameters of L* and b*. The L* parameter denotes lightness (from 0 for black to 100 for white), and b* is negative for blue and positive for yellow.

2.2. Reflectance Measurement Acquisition and Selection of Spectral Reflectance Indices

The spectra of fruit samples for each type of mango and strawberry were collected with a handheld spectrometer (tec5 AG, Oberursel, Germany), which could measure light with a wavelength from 302 nm to 1148 nm with a final spectral interval of 2 nm. On cloud-free days between 11 and 13 h GMT, spectra were acquired from different ripening degrees of fruits under sun radiation. Fruit samples were put above a black sheet, and the spectrometer was held vertically at a nadir position roughly 25 cm above the water surface with a scanning area of 0.03 m^2. The final reflaction curve for each fruit was the average of the spectral reflectance of the three different places with 10 scans. The reflaction was determined by correcting spectrometer results with a calibration factor obtained from a white reference standard (A polytetrafluoroethylene white Spectral on reflectance panel). Processed spectra were then used to derive different SRIs. Table 1 lists some of the most widely used SRIs as well as the method for calculating them, along with references. Sixteen SRIs, including the four most widely used SRIs and twelve newly developed two-band (2-D) SRIs, were illustrated (Table 1). Statistics were displayed on contour maps as determination coefficients (R^2) between several measured parameters (SRIs) (Figures 1 and 2). These indices were calculated by integrating potentials at any two wavelengths in a spectrum region ranging from 302 to 1148 nm.

$$RSI = R_1/R_2 \quad (1)$$

R_1 and R_2 refer to the values of spectral reflectance at various wavelengths.

Table 1. Published and newly constructed two-band spectral reflectance indices with their formulas and relevant references (Ref.)

SRIs	Formula	Ref.
Published index		
Greenness index (GI)	R_{554}/R_{677}	[38]
Pigment-Sensitive Ripening Monitoring Index (PSRMI)	$(R_{750} - R_{678})/R_{550}$	[39]
Anthocyanin index (NAI)	$(R_{760}-R_{720})/(R_{760} + R_{720})$	[40]
Normalized difference vegetation index (NDVI)	$(R_{780} - R_{670})/(R_{780} + R_{670})$	[41]
Newly Ratio Spectral Index (RSI)		
$RSI_{666,636}$	R_{666}/R_{636}	This study
$RSI_{660,620}$	R_{660}/R_{620}	
$RSI_{670,610}$	R_{670}/R_{610}	
$RSI_{618,602}$	R_{618}/R_{602}	
$RSI_{640,590}$	R_{640}/R_{590}	
$RSI_{970,590}$	R_{970}/R_{590}	
$RSI_{764,766}$	R_{764}/R_{766}	
$RSI_{768,770}$	R_{768}/R_{770}	
$RSI_{772,762}$	R_{772}/R_{762}	
$RSI_{766,764}$	R_{766}/R_{764}	
$RSI_{760,858}$	R_{760}/R_{858}	
$RSI_{970,1000}$	R_{970}/R_{1000}	

2.3. Machine learning methods for mango and strawberry quality assessment

Three machine learning models, like an artificial neural network (ANN), a random forest (RF), and a decision tree (DT), were created using the Python scikit-learn library and Spyder software. These models are used to predict mango and strawberries' biochemical parameters. The input data for these models are spectral indices selected based on their correlation above a predetermined threshold. This study randomly divided the dataset into training (70%) and testing (30%) sets. The random state was determined as 0, which controls randomness and sampling variables to split the nodes. The model predetermines its hyper-parameters before training, instead of learning them from the data. It plays a crucial role in determining the model's performance, as noted by Hossain and Timmer [42]. Cross-validation with a 5-fold size and the grid-search method were employed on the training samples to test different hyperparameter combinations. This testing is employed to select optimal hyperparameters that achieve the best performance for three ML models based on the lowest MSE and the highest R^2 value. Schematic diagram of the models was presented in Figure 3.

2.3.1. Artificial Neural Network (ANN)

ANN models were developed consisting of input layers, hidden layers, and output layers. Each layer consists of neurons. The neuron known as perceptron is similar to multiple linear regression. In this study, stochastic gradient descent (SGD) is selected as the optimization method, as shown in equation (2). This method is used to adjust the weights and reduce the variation between the expected values and the real laboratory estimated values, as Oymak [43] pointed out. The hyperparameters that need tuning include the number of neurons for each hidden layer, the number of hidden layers, and the activation function that is shown in Table 2. The structure of the ANN model is determined by trial and error, as mentioned by Mijwel [44].

$$\theta_{j+1} \coloneqq \theta_j - \alpha.\left(Y_a{}^{(i)} - Y_p{}^{(i)}\right).x_j{}^{(i)} \qquad \ldots\ldots\ldots\ldots\ldots \qquad (2)$$

θ_{j+1}: Weights of the next iteration, θ_j: Weights of the current iteration, α: Learning rate, $x_j{}^{(i)}$: input feature, $Y_a{}^{(i)}$: Actual value, $Y_p{}^{(i)}$: Predict value.

Table 2. Activation Function, as stated in Sharma *et al.* [45].

Name	Equations
Hyperbolic Tangent (Tanh)	$f(x) = \frac{(e^x - e^{-x})}{(e^x + e^{-x})}$
Logistic (Sigmoid)	$f(x) = \frac{1}{1 + e^{-x}}$
Rectified Linear Unit (ReLU)	$f(x) = \max(0, x)$
Linear (Identify)	$f(x) = x$

2.3.2. Random Forest (RF)

Random forest is a widely used machine learning algorithm employed for classification or regression tasks. It utilizes a combination of multiple decision trees to enhance prediction accu-

racy. At its core, a random forest comprises an ensemble of decision trees generated from random subsets of the available data. In this study, three key hyperparameters were considered: the number of trees in the forest, the maximum depth of individual trees, and the criterion function. The primary purpose of employing random forests is to overcome the overfitting problem commonly associated with individual decision trees. By aggregating predictions from multiple trees, the random forest output is evaluated by averaging the results, as suggested by Breiman [46]. This ensemble technique significantly improves the overall performance and the model's ability to generalize to unseen data.

2.3.3. Decision Tree (DT)

The decision tree algorithm is made up of leaf nodes, decision nodes, branches, and a root node. It is organized like a tree. The root node starts the tree, whereas the decision nodes make decisions that decide the path, which moves from one node to another. The decision nodes end with the leaf nodes, Han et al. [47]. Regression rules are easily created using decision trees. Because the DT doesn't require parameter setting or domain expertise, it is appropriate for exploratory knowledge discovery. During training, hyperparameter optimization was carried out, and the optimal parameters were used to create the top-level model Xia et al. [48]. In this study, two key hyperparameters were taken into account during training to optimize the decision tree model: the maximum depth of the tree, which varied from 1 to 10, and the criterion used to judge the quality of a split.

2.4. Models Evaluation

In order to assess the efficacy of the three ML models, the coefficient of determination (R^2) and the mean square error (MSE) equations were employed [49,50]. The variables utilized in these equations are specified as follows: "Yact" represents the actual laboratory-measured value, "Yp" represents the predicted value, "Yave" represents the average value, and "T" represents the dataset used in both the training and testing stages.

$$\mathrm{MSE} = \frac{1}{N}\sum_{i=1}^{T}(Y_{act} - Y_{p})^2 \quad \text{.............} \quad (3)$$

$$R^2 = \frac{\sum(Y_{act} - Y_{p})^2}{\sum(Y_{act} - Y_{ave})^2} \quad \text{.............} \quad (4)$$

3. Results and Discussion

3.1. Effect of Ripening Stages on Quality Parameters of Mango Fruits

The mango fruit quality parameters have a significant impact during the ripening stages. The SPAD values and firmness of the mango fruit exhibited a decline. Conversely, the TSS exhibited a progressive increase in its values across the ripening process. Table 3 displays significant differences in the quality parameters of mango fruit at various ripening stages. The SPAD values vary from 0.00 to 36.00 (dimensionless), the TSS values range from 8.2 to 21.9 (°Brix), and the firmness values range from 0.00 to 42.5 (N). The reduction in chlorophyll content in fruits can serve as a crucial indicator of fruit quality [51]. According to Medlicott et al. [52], the mango's chlorophyll content decreases as it ripens, as indicated by SPAD measures. The breakdown of chlorophyll pigments and the emergence of other pigments during fruit maturation are responsible for this decrease. Lizada [53] explains that chloroplasts undergo a transformation, containing

yellow or red pigments in the process. The unripe stage of mango fruits has high chlorophyll content values, induced by higher SPAD measures (27.56), which then decreases at the ripe stage to 6.23. The chlorophyll content of mango fruit decreases at the overripe stage, which leads to a decrease in spade values of 0.06 (Table 3). The gradual decrease in SPAD values corresponds to the color transition of the mango peel from green to yellow or orange, which indicates the ripeness of the fruit. The results of our study are consistent with what Elsayed et al. [25] found in their study. The TSS is an important index for fruit ripening because it indicates the acceptance of nutrients and the economic worth of fruit in trading. Our findings revealed that the overripe stage recorded the highest TSS value (19.93 °Brix), while the ripe stage recorded 17.81 °Brix. Table 3 displays the lowest TSS value (11.98 °Brix) during the unripe stage. The rise in TSS content can be attributed to the enzymatic transformation of intricate carbohydrates into more basic sugars, aided by the phosphorylase enzyme throughout the maturation procedure, as corroborated by Palafox-Carlos et al. [54]. According to Appiah et al. [55], the increase in TSS during the ripening phase validates the anticipated sweetening effect. Our results support the rising trend of TSS during mango ripening, which is consistent with earlier research by Subedi et al. [56], Islam et al. [57] and Nambi et al. [58]. Meanwhile, the firmness of the fruit is commonly used as an indicator of fruit quality. Our investigation's results revealed that the highest firmness value was achieved at the unripe stage, reaching 24.68 N. At the ripe stage, we observed a clear decrease in firmness value with a 2.24 N. The firmness saw a drop to its lowest value during the overripe stage, with a value of 0.78 N (Table 3). The gradual decrease in firmness can be attributed to the release of water moving from the peel to the pulp. Furthermore, enzymatic activity enhances the degradation of cell wall polysaccharides and the transformation of starch into more basic compounds during fruit ripening [59]. Our findings agree with the observations made by Robles-Sánchez et al. [60] and Palafox-Carlos et al. [54].

3.2 Effect of Ripening Stages on Quality Parameters of Strawberry Fruits

The quality parameters of strawberry fruits are clearly affected during fruit ripening. L*, b*, and the firmness of the fruits showed a constant decrease. In contrast, TSS showed a continuous increase during strawberry fruit ripening. Table 4 shows the significant differences between the fruit quality parameters' values at different maturity stages. There is also a wide range of values for all quality parameters at different fruit ripening stages, as shown in Table 4. The L* value ranged from 29.84 to 43.32, the b* values ranged from 13.21 to 26.92, the TSS value ranged from 7.50 to 12.10, and the firmness value ranged from 3.28 to 7.92. Table 4 displays the findings of our study, which indicate that the maximum L* value (39.62) was found during the unripe stage, followed by 35.43 during the ripe stage, and the lowest value (31.35) was found during the overripe stage. These findings indicate that strawberry fruit's brightness diminishes as it ripens. In other words, as fruit ripens, it becomes darker. These results are consistent with the results of Saad et al. [5]. The highest b* value (24.46) was recorded during the unripe stage, followed by 21.80 during the ripe stage. The lowest b* values (21.80) were observed during the overripe stage (Table 4). The gradual decrease in b* values during ripening processes is attributed to the fact that the fruit turns red as a result of the synthesis of pigments such as carotenoids, anthocyanins, and flavonoids, according to Winarno [61]. This change in color is used as an indicator of the ripening process of strawberry fruits, as pointed out by Cahyono [62]. These results are in agreement with Janurianti et al. [63] and Tsormpatsidis et al. [64]. TSS is an important indicator to determine fruit maturity. In our study, we found significant differences in TSS values during different ripening stages. The highest TSS value is 10.42 °Brix during the overripe phase. During the ripe stage, the value was detected as 9.33 °Brix. The lowest value of 8.53 °Brix was observed during the unripe stage (Table 4). This gradual increase during the ripening processes of strawberry fruit can be attributed to the increased concentration of sugars and organic acids [65,66]. In addi-

tion, Ullah [67] and Bhatti [68] also pointed out that with the progress of fruit ripening, the sugar content increases as a result of the hydrolysis of sucrose, which leads to an increase in TSS. With the transition of the fruit from the unripe stage to the overripe stage, the moisture content in the strawberry fruit decreases and, accordingly, the concentration of Tess becomes higher and the increase appears, as Martínez et al. [69] mentioned. The gradual increase is consistent with previous studies by Rahman et al. [70], and Janurianti et al. [63]. The firmness parameter plays an important role in determining the suitability of the fruit for commercialization. It is an indicator of mechanical resistance Gunness et al. [71]. We discovered significant differences between the values of firmness at different ripening stages. During the unripe stage, the highest firmness value is 7.09 N. The ripening stage has a value of 5.92 N. The lowest values of 3.84 N are recorded during the overripe stage (Table 4). The tendency of this parameter to decrease during the ripening process of strawberry fruit corresponds to de Jesús Ornelas-Paz et al. [72].

Table 3. Statistical summary of several biochemical parameters for mango fruits.

Mango Fruits												
Stage	Unripe				Ripe				Overripe			
Parameters	Min	Max	Mean	SD	Min	Max	Mean	SD	Min	Max	Mean	SD
SPAD	19.50	36.00	27.56a	4.53	0.20	18.50	6.23b	5.54	0.00	0.20	0.06c	0.07
TSS (°Brix)	8.20	15.9	11.98c	2.24	16.60	19.00	17.81b	0.74	17.80	21.90	19.93a	1.07
Firmness (N)	5.90	42.5	24.68a	9.50	1.30	5.85	2.24b	1.29	0.00	1.25	0.78b	0.39

Table 4. Statistical summary of several biochemical parameters for strawberry fruits.

Strawberry Fruits												
Stage	Unripe				Ripe				Overripe			
Parameters	Min	Max	Mean	SD	Min	Max	Mean	SD	Min	Max	Mean	SD
L*	35.20	43.32	39.62a	2.18	31.44	42.24	35.43b	2.61	29.84	33.47	31.35c	1.04
B*	22.19	26.92	24.46a	1.42	19.38	24.87	21.80b	1.84	13.21	17.83	15.52c	1.44
TSS (°Brix)	7.50	9.70	8.53c	0.47	8.50	10.00	9.33b	0.51	9.70	12.10	10.42a	0.59
Firmness (N)	5.63	7.92	7.09a	0.69	4.47	6.97	5.92b	0.69	3.28	4.53	3.84c	0.36

3.3 Variation of SRIs at Different Ripening Degrees for Mango and Strawberry Fruits

The spectral reflectance of fruit surfaces can reveal important information about alterations in the fruit's biochemical parameters. These alterations result in variations in spectral reflectance indices (SRIs) across specific wavelengths, according to Sirisomboon [14]. The biochemical parameters of strawberry and mango fruit are marked by SRIs in Tables 5 and 6, respectively. These SRIs values show significant changes across different ripening stages, attributed to significant variations in biochemical parameters. For mango fruit, quantitative analysis shows a significant variation in the mean values of mango fruit's biochemical parameters. The SPAD value ranged from 0.00 to 36.00, the TSS values ranged from 8.20 to 21.90, and the firmness value ranged from 0.00 to 42.50. These alterations are followed by corresponding changes in the mean values of indices, including $RSI_{764,766}$, $RSI_{768,770}$, $RSI_{772,762}$, and $RSI_{766,764}$, with values ranging from 0.992 to 1.009, 0.991 to 1.003, 0.974 to 1.039, and 0.981 to 1.024, as indicated in Table 5. All SRIs values for mango fruit have a significant difference across various ripening stages. For strawberry fruits, quantitative analysis reveals significant variation in quality parameters. These parameters such as, L*, b*, TSS, and firmness value ranged from 29.84 to 43.32, 13.21 to 26.92, 7.50 to 12.10, and 3.28 to 7.92, respectively. These changes are attributed to alterations in the mean values of indices, namely $RSI_{666,636}$, $RSI_{660,620}$, $RSI_{670,610}$, and $RSI_{618,602}$, with values ranging from 0.715 to 1.328, 0.779 to 1.955, 0.665 to 2.694, and 1.005 to 1.740, as shown in Table 6. Most of the SRIs values for strawberry fruit's quality parameters show a significant difference during different ripening stages. However, the two indices ($RSI_{970,590}$ and PRMI) show no significant difference between the unripe and ripe stages. The NAI values also show no significant difference between the three ripening stages. In general, the gradual increase or decrease in SRIs values during different ripening stages can be attributed to changes in the quality parameters of fruits Elsayed et al. [51].

Table 5. Statistical summary of several spectral reflectance indices for mango fruits.

Stage	Unripe				Ripe				Overripe			
Parameters	Min	Max	Mean	SD	Min	Max	Mean	SD	Min	Max	Mean	SD
$RSI_{764,766}$	1.001	1.009	1.004a	0.002	0.997	1.000	0.998b	0.001	0.992	0.997	0.995c	0.001
$RSI_{768,770}$	0.997	1.003	1.000a	0.002	0.994	0.997	0.996b	0.001	0.991	0.995	0.993c	0.001
$RSI_{772,762}$	0.974	1.004	0.990c	0.008	1.004	1.018	1.012b	0.004	1.017	1.039	1.026a	0.006
$RSI_{766,764}$	0.981	1.010	0.997c	0.008	1.011	1.024	1.018b	0.004	1.024	1.046	1.032a	0.006
$RSI_{760,858}$	1.101	1.189	1.146a	0.021	1.076	1.141	1.100b	0.016	1.035	1.111	1.073c	0.021
$RSI_{970,1000}$	0.927	0.980	0.947a	0.014	0.898	0.942	0.922b	0.012	0.904	0.931	0.914c	0.007
GI	1.262	3.141	2.526a	0.458	0.890	3.205	1.990b	0.667	0.500	2.818	1.120c	0.669
PRMI	0.874	1.671	1.349a	0.205	0.431	1.625	1.124b	0.301	-0.067	1.394	0.563c	0.472
NAI	-0.010	0.106	0.060a	0.034	-0.014	0.115	0.031b	0.034	-0.022	0.090	0.003c	0.024
$NDVI_{780,670}$	0.367	0.713	0.624a	0.086	0.179	0.716	0.514b	0.154	-0.001	0.662	0.256c	0.217

Table 6. Statistical summary of several spectral reflectance indices for strawberry fruits.

Stage	Unripe				Ripe				Overripe			
Parameters	Min	Max	Mean	SD	Min	Max	Mean	SD	Min	Max	Mean	SD
$RSI_{666,636}$	0.715	0.877	0.77c	0.040	0.759	0.887	0.84b	0.036	1.062	1.328	1.17a	0.075
$RSI_{660,620}$	0.799	1.088	0.88c	0.071	0.901	1.117	1.01b	0.056	1.373	1.955	1.62a	0.143
$RSI_{670,610}$	0.665	1.092	0.78c	0.106	0.793	1.121	0.95b	0.087	1.608	2.694	2.04a	0.268
$RSI_{618,602}$	1.005	1.271	1.08c	0.070	1.086	1.293	1.17b	0.053	1.457	1.740	1.57a	0.075
$RSI_{640,590}$	0.998	1.948	1.26c	0.245	1.286	2.079	1.59b	0.204	2.892	4.689	3.57a	0.483
$RSI_{970,590}$	0.801	2.093	1.29b	0.322	1.406	2.330	1.68b	0.247	2.379	5.667	3.93a	0.832
GI	0.294	1.065	0.68a	0.187	0.234	0.628	0.39b	0.119	0.072	0.142	0.10c	0.019
PRMI	1.075	3.952	1.97b	0.809	1.617	4.285	2.74b	0.719	3.618	11.919	6.56a	2.015
NAI	0.003	0.294	0.016a	0.007	0.011	0.025	0.019a	0.004	0.006	0.028	0.016a	0.006
$NDVI_{780,670}$	0.029	0.445	0.40a	0.041	0.317	0.422	0.36b	0.027	0.183	0.337	0.28c	0.047

3.4 Evaluation of Spectral Reflectance Indices (SRIs) to Assess the Quality Parameters

The effectiveness of both previously published and newly devised SRIs in detecting biochemical parameters in mango and strawberry fruits was assessed in this study. Figures 4a and 4b exhibit the findings, illustrating that the newly developed SRIs are superior in assessing biochemical parameters. Nevertheless, the majority of published SRIs also demonstrated correlations with biochemical parameters. For mango fruit, the published SRIs showed R^2 values ranging from 0.29 to 0.38 for SPAD, 0.30 to 0.41 for TSS, and 0.22 to 0.31 for firmness. The R^2 values exhibited relatively low values for all mango quality parameters when employing the published SRIs, as depicted in Figure 4a. The newly developed SRIs obtained R^2 values ranging from 0.71 to 0.91 for SPAD, 0.62 to 0.88 for TSS, and 0.65 to 0.84 for firmness. The mango fruit's biochemical parameters had the highest R^2 values with newly developed SRIs, which were derived from the near-infrared (NIR) region. For example, the $RSI_{764,766}$ and $RSI_{768,770}$ displayed R^2 values of 0.91 and 0.91 for SPAD, 0.88 and 0.87 for TSS, and 0.84 and 0.85 for firmness, respectively, as seen in Figure 4a. For strawberry fruit, the published SRIs presented R^2 values ranging from 0.02 to 0.70 for L*, 0.00 to 0.66 for b*, 0.00 to 0.60 for TSS, and 0.00 to 0.69 for firmness. The R^2 values were low with all quality parameters when using the published SRIs, particularly with the NAI index (Figure 4b). Conversely, the newly developed SRIs yielded R^2 values ranging from 0.60 to 0.67, 0.71 to 0.84, 0.57 to 0.65, and 0.69 to 0.79 for L*, b*, TSS, and firmness, respectively. The newly developed SRIs, extracted from the red region in the visible spectrum, showed the best R^2 values with the biochemical parameters of strawberry fruit. For example, $RSI_{666, 636}$ and $RSI_{660, 620}$ showed R^2 values of 0.67 and 0.66 for L*, 0.84 and 0.83 for b*, 0.63 and 0.63 for TSS, and 0.79 and 0.78 for firmness, respectively, as shown in Figure 4b.

Several studies have highlighted the potential of using spectral reflectance indices to remotely and non-destructively assess different biochemical parameters in fruit [5,73–75]. For instance, Elsayed et al. [25] reported that the SRIs produced from visible or near-infrared regions are usefull in determining the soluble solids content and SPAD value of mango fruits. Tijskens et al. [76] and Wei et al. [77] revealed that variations in pigment components for strawberry fruit like anthocyanin and chlorophyll could be linked to variations in spectral density in the 550–900 nm region. Furthermore, Yahaya et al. [78] reported that the red wavelengths (600-700 nm) are efficiently absorbed by mango pigments. These wavelengths and the assessments of mango fruit hardness, acidity, and soluble solids content (SSC) showed the strongest relationships, as a consequence. These studies demonstrate how spectral reflectance indices can be used to quickly and non-destructively evaluate fruit quality by estimating and evaluating a variety of biochemical characteristics.

3.5. ML-Models Performance for Mango Quality Assessment

Three machine learning (ML) models have demonstrated remarkable accuracy in predicting important parameters for mango, namely TSS, SPAD, and firmness, as illustrated in Table 7 across both the training and testing phases. The findings of the testing phase are displayed in Figure 5. The artificial neural network (ANN-TSS) model obtained an R^2 value of 0.92 and 0.93 for TSS prediction, with corresponding MSE values for the training and testing phases of 1.13 and 0.52, respectively. The ANN-TSS model was built using a single hidden layer that had five neurons (Figure 6a). This model also employed the logistic activation function and underwent 500 iterations. The random forest (RF-TSS) model surpassed even greater accuracy, achieving an R^2 value of 0.98 and 0.93, along with an MSE of 0.30 and 0.51 for the training and testing phases, respectively. The RF-TSS model consisted of 15 trees and a maximum depth of 4. This model used the squared error as the criterion function. Moreover, the decision tree (DT-TSS) model performed admirably, showing R^2 values of 0.95 and 0.88 for the training and testing phases, respectively, along with MSE values of 0.70 and 0.87. The

DT-TSS model had a maximum depth of 3. This model used the squared error as the criterion function. These results demonstrate how well these ML models can predict mango fruit's TSS. In terms of SPAD prediction, the artificial neural network (ANN-SPAD) model performed exceptionally well, with R^2 values of 0.99 and 0.98 and MSE values of 0.83 and 1.34 for the training and testing phases, respectively. The ANN-SPAD model's construction had five neurons in each of the two hidden layers, as depicted in Figure 6b. This model went through 700 iterations using the relu activation function. Additionally, the random forest (RF-SPAD) model showed impressive accuracy, giving MSE values of 1.53 and 0.25 and an R^2 value of 0.99 and 0.98 for the testing and training phases, respectively. Comprising 20 trees with a maximum depth of 5, the RF-SPAD model employed the squared error as the criterion function. In contrast, the decision tree (DT-SPAD) model exhibited an R^2 value of 0.99 and 0.98, accompanied by MSE values of 0.02 and 2.14 for the training and testing phases, respectively. Constructed with a maximum depth of 5 and utilizing the squared error as the criterion function. These impressive results further emphasize the efficacy of these machine learning models in forecasting SPAD values, underscoring their significance in precise mango quality assessment.

In terms of firmness prediction, the artificial neural network (ANN-Firmness) model performed exceptionally well, with R^2 values of 0.99 and 0.98 and matching MSE values of 1.28 and 1.22 for the training and testing phases, respectively. Four neurons, each in two hidden layers, were used to construct the ANN- Firmness (Figure 6c). This model used the relu activation function. Its performance was optimized through 700 iterations. The random forest (RF-Firmness) model provides accuracy in predicting mango fruit's firmness, with an astounding R^2 value of 0.99 and MSE values of 0.49 and 0.59 for the training and testing phases, respectively. The RF-Firmness model consisted of 20 trees with a maximum depth of 5. The squared error served as the criteria function for this model. With an R^2 value of 0.99 and 0.99 and matching MSE values of 0.03 and 0.96, throughout the training and testing stages, respectively. The decision tree (DT- Firmness) model demonstrated exceptional performance. With a maximum depth of 5 and the squared error served as the criterion function. These outcomes show how well machine learning models predict the mango fruit's firmness.

Table 7. Performance of different ML models for prediction of TSS, SPAD, and firmness for Mango after training and testing phase.

Target Variables	Models	Optimal Input Variables	Training		Testing	
			R^2	MSE	R^2	MSE
TSS	ANN-TSS	$RSI_{764,766}$ - $RSI_{768,770}$ - $RSI_{772,762}$ - $RSI_{766,764}$ - $RSI_{760,858}$ - $RSI_{970,1000}$	0.9242	1.13	0.9288	0.52
	RF-TSS	$RSI_{764,766}$ - $RSI_{768,770}$ - $RSI_{772,762}$ - $RSI_{766,764}$ - $RSI_{760,858}$ - $RSI_{970,1000}$ – GI – PRMI – $NDVI_{780,670}$	0.9800	0.30	0.9300	0.51
	DT-TSS	$RSI_{764,766}$ - $RSI_{768,770}$ - $RSI_{772,762}$ - $RSI_{766,764}$ - $RSI_{760,858}$	0.9531	0.70	0.8819	0.87
SPAD	ANN-SPAD	$RSI_{764,766}$ - $RSI_{768,770}$ - $RSI_{772,762}$ - $RSI_{766,764}$	0.9951	0.83	0.9847	1.34
	RF-SPAD	$RSI_{764,766}$ - $RSI_{768,770}$ - $RSI_{772,762}$ - $RSI_{766,764}$	0.9985	0.25	0.9826	1.53
	DT-SPAD	$RSI_{764,766}$ - $RSI_{768,770}$ - $RSI_{772,762}$ - $RSI_{766,764}$	0.9999	0.02	0.9756	2.14
Firmness	ANN-Firmness	$RSI_{764,766}$ - $RSI_{768,770}$ - $RSI_{772,762}$ - $RSI_{766,764}$ - $RSI_{760,858}$ - $RSI_{970,1000}$	0.9925	1.28	0.9814	1.22
	RF- Firm-	$RSI_{764,766}$ - $RSI_{768,770}$ - $RSI_{772,762}$ -	0.9972	0.49	0.9909	0.59

ness	$RSI_{766,764}$ - $RSI_{760,858}$ - $RSI_{970,1000}$				
DT- Firmness	$RSI_{764,766}$ - $RSI_{768,770}$ - $RSI_{772,762}$ - $RSI_{766,764}$ - $RSI_{760,858}$ - $RSI_{970,1000}$	0.9998	0.03	0.9853	0.96

The complete names of the abbreviated hyperspectral indices can be found in Table 1.

3.6. ML-Models Performance for Strawberry Quality Assessment

Table 8 and Figure 7 illustrate how well ML models have predicted quality parameters for strawberries. For the L* prediction, the artificial neural network (ANN-L*) model demonstrated remarkable accuracy with R^2 values of 0.87 and 0.75 and corresponding MSE values of 2.20 and 3.07, during the training and testing phases, respectively. As seen in Figure 8a, the ANN-L* model was created with two hidden layers, each containing four neurons. It ran through 500 iterations using the relu activation function. The random forest (RF-L*) model achieved high accuracy, obtaining R^2 values of 0.88 and 0.74 and MSE values of 1.84 and 3.17 for the training and testing phases, respectively. This model used squared error as a criterion function. It consists of 15 trees with a maximum depth of 2. Furthermore, during the training and testing stages, the decision tree (DT-L*) model showed excellent performance, with R^2 values of 0.91 and 0.74 and MSE values of 1.52 and 3.27, respectively. This model was built with a maximum depth of 2. It used the squared error as the criteria function. These results confirm the models' capacity to accurately predict L*. For b* prediction, the artificial neural network (ANN-b*) model exhibited exceptional performance, with R^2 values of 0.91 and 0.91 for the training and testing phases, respectively. The MSE values that were consistent were 1.43 and 1.38. The ANN-b* model was developed using one hidden layer, each of which contained seven neurons (Figure 8b). The relay activation function was employed to optimize its performance over 700 iterations. In the same vein, the random forest (RF-b*) model demonstrated exceptional accuracy, achieving R^2 values of 0.91 and 0.84 for the training and assessment phases, respectively, with MSE values of 1.42 and 2.40. The RF-b* model consisted of 10 trees with a maximum depth of 2. It employs the squared error as the criterion function. In contrast, the decision tree (DT-b*) model achieved an R^2 value of 0.78 and 0.79, accompanied by MSE values of 3.68 and 3.16 during the training and testing phases, respectively. The DT-b* model was built with a maximum depth of 1, and the squared error was used as the criterion function. These remarkable outcomes demonstrate the effectiveness of these machine learning models in precisely predicting b* values. These ML models underline their importance in accurately predicting the quality of strawberry fruit. For TSS prediction, the artificial neural network (ANN-TSS) model performed exceptionally well, with R^2 values of 0.76 and 0.58 and MSE values of 0.20 and 0.37, for the training and testing phases, respectively. Five neurons make up its single hidden layer architecture (Figure 8c). The Tanh activation function was employed. For performance optimization, it went through 500 iterations. Likewise, the random forest (RF-TSS) model demonstrated excellent accuracy, achieving training and testing phases' R^2 values of 0.87 and 0.86 along with MSE values of 0.11 and 0.13. The RF-TSS model is made up of 20 trees with a maximum depth of 5. It used the squared error as the criteria function. On the other hand, throughout the training and testing stages, the decision tree (DT-TSS) model obtained an R^2 value of 0.75 and 0.80, with MSE values of 0.21 and 0.18, respectively. Using the squared error as the criterion function. The DT-TSS model was built with a maximum depth of 2. These remarkable findings demonstrate the effectiveness of these machine learning models in precisely predicting TSS levels. In terms of firmness prediction, the artificial neural network (ANN-Firmness) model performed exceptionally well, showing R^2 values of 0.81 and 0.84, respectively, for training and testing phases. According to the corresponding MSE values of 0.39 and 0.36, the model can accurately measure firmness. The ANN-Firmness model was built using two hidden layers, each with three neurons (Figure 8d). Tanh activation function was employed. Its performance was optimized through 700 iterations. In a similar vein, the random forest (RF-Firmness) model showed excellent accuracy, with R^2 values for the training and testing phases of 0.85 and 0.80, respectively, and MSE values of 0.31 and 0.44. The RF-Firmness model is made up 20 trees with a

maximum depth of 2. It used the squared error as the criteria function. Finally, the decision tree (DT-Firmness) model achieved R^2 with 0.86 and 0.80, the corresponding MSE values of 0.29 and 0.81, for the training and testing phases, respectively. The DT- Firmness model has a maximum depth of 2. The criteria function for this model was the squared error. These remarkable results show how accurate and reliable these ML models are at predicting firmness. Their ability to generate reliable judgments emphasizes their significance in precisely evaluating strawberry quality. Mithun et al. [79] used spectral reflectance data points taken from the spectral range of 350 nm to 1050 nm, combined with RF and DT models, for the detection of artificially ripened mango. Their results exhibited high accuracy rates of 97.22% for RF and 98.91% for DT. Munawar [80] reported that the optimal models for mango quality parameter prediction were achieved by coupling ANN with NIR. Zhang et al. [81] employed NIR data collected during the mango ripening season incorporated with an ANN model for a mango fruit's TSS detection, resulting in a R^2 of 0.9055 and an RMSE of 0.2192.

Table 8. Performance of different ML models for prediction of L, b, TSS, and firmness for Strawberry after training and testing phase.

Target Variables	Models	Optimal Input Variables	Training		Testing	
			R^2	MSE	R^2	MSE
L*	ANN-L*	$RSI_{666,636}$ - $RSI_{660,620}$ - $RSI_{670,610}$ - $RSI_{618,602}$ - $RSI_{640,590}$ - $RSI_{970,590}$ – GI – PRMI – $NDVI_{780,670}$	0.8659	2.20	0.7525	3.07
	RF-L*	$RSI_{666,636}$ - $RSI_{660,620}$ - $RSI_{670,610}$ - $RSI_{618,602}$ - $RSI_{640,590}$ - $RSI_{970,590}$ - GI	0.8879	1.84	0.7443	3.17
	DT-L*	$RSI_{666,636}$ - $RSI_{660,620}$ - $RSI_{670,610}$ - $RSI_{618,602}$ - $RSI_{640,590}$ - $RSI_{970,590}$ - GI	0.9073	1.52	0.7360	3.27
b	ANN-b*	$RSI_{666,636}$ - $RSI_{660,620}$ - $RSI_{670,610}$ - $RSI_{618,602}$ - $RSI_{640,590}$ - $RSI_{970,590}$ - GI	0.9139	1.43	0.9102	1.38
	RF-b*	$RSI_{666,636}$ - $RSI_{660,620}$ - $RSI_{670,610}$ - $RSI_{618,602}$ - $RSI_{640,590}$ - $RSI_{970,590}$ – GI – PRMI – $NDVI_{780,670}$	0.9148	1.42	0.8438	2.40
	DT-b*	$RSI_{666,636}$ - $RSI_{660,620}$ - $RSI_{670,610}$ - $RSI_{618,602}$ - $RSI_{640,590}$ - $RSI_{970,590}$ - GI	0.7787	3.68	0.7942	3.16
TSS	ANN-TSS	$RSI_{666,636}$ - $RSI_{660,620}$ - $RSI_{670,610}$ - $RSI_{618,602}$ - $RSI_{640,590}$ - $RSI_{970,590}$ – GI – PRMI – $NDVI_{780,670}$	0.7541	0.20	0.5836	0.37
	RF-TSS	$RSI_{666,636}$ - $RSI_{660,620}$ - $RSI_{670,610}$ - $RSI_{618,602}$ - $RSI_{640,590}$ - $RSI_{970,590}$ – GI – PRMI – NAI - $NDVI_{780,670}$	0.8684	0.11	0.8568	0.13
	DT-TSS	$RSI_{666,636}$ - $RSI_{660,620}$ - $RSI_{670,610}$ - $RSI_{618,602}$ - $RSI_{640,590}$ - $RSI_{970,590}$ – GI – PRMI – $NDVI_{780,670}$	0.7528	0.21	0.8002	0.18
Firmness	ANN-Firmness	$RSI_{666,636}$ - $RSI_{660,620}$ - $RSI_{670,610}$ - $RSI_{618,602}$ - $RSI_{640,590}$ - $RSI_{970,590}$ – GI – PRMI – $NDVI_{780,670}$	0.8135	0.39	0.839	0.36
	RF-Firmness	$RSI_{666,636}$ - $RSI_{660,620}$ - $RSI_{670,610}$ - $RSI_{618,602}$ - $RSI_{640,590}$ - $RSI_{970,590}$ - GI	0.8532	0.31	0.8004	0.44
	DT-Firmness	$RSI_{666,636}$ - $RSI_{660,620}$ - $RSI_{670,610}$	0.8609	0.29	0.8083	0.43

The complete names of the abbreviated hyperspectral indices can be found in Table 1.

Devassy and George [36] showed the effectiveness of using spectral reflectance with an RF model to predict the strawberry fruit's firmness in a rapid, non-destructive method with an R^2 of prediction of 0.90 and 0.94 for training and testing, respectively. Amoriello et al. [82] investigated the feasibility of employing ANN models in conjunction with spectral reflectance in the wavelength range of 400–1000 nm to forecast strawberry quality parameters. Prediction models were observed with an R^2 = 0.81 for firmness, 0.96 for TSS, 0.88 for titrata-

ble acidity, and 0.95 for dry matter. ElMasry et al. [83] used color features extracted from strawberry images, which contained the brightness values, for fruit firmness prediction using ANN. The efficiency of the model in classifying fruits was 92.88%.

5. Conclusions

This study was to evaluate the biochemical parameters of mango and strawberry fruits at various ripening stages. This was done by utilizing a combination of established and newly developed SRIs in conjunction with ML models, including ANN, RF, and DT. Our results revealed significant differences in the fruit's biochemical parameters across various ripening stages. The published SRIs showed strong correlations with the selected parameters, but the newly developed SRIs were more effective at monitoring the various biochemical parameters. The integration of SRIs with diverse ML models proved to be a successful strategy for precisely estimating biochemical parameters. For mango biochemical parameters prediction, the ANN models demonstrated R^2 values ranging from 0.92 to 1.00 and from 0.93 to 0.98 for training and testing, respectively. On the other hand, the RF models exhibited R^2 values ranging from 0.98 to 1.00 and from 0.93 to 0.99 during training and testing, respectively. The DT models showed highly performance with R^2 values ranging from 0.95 to 1.00 and 0.88 to 0.99 for training and testing phases. For strawberry's biochemical parameters prediction, the ANN models achieved R^2 values between 0.75 and 0.91 and between 0.58 and 0.91 during training and testing phases, respectively. On the other hand, RF models showed R^2 values between 0.85 and 0.91 during training and between 0.74 and 0.86 during testing. The DT models demonstrated good results, with R^2 values ranging from 0.75 to 0.91 for the training set and 0.74 to 0.81 for the testing set. These findings underscore the effectiveness of combining SRIs with ML models, particularly ANN, RF, and DT, for accurate prediction of biochemical parameters in mango and strawberry fruits.

Author Contributions: Conceptualization, S.E.; methodology, S.E.; H.G.; and N.G.A.; software, S.E.; H.G.; M.S.A.; M.M.; and A.E.E.; validation, S.E.; N.G.A.; and H.G.; formal analysis, S.E.; N.G.A.; and H.G.; investigation, S.E.; A.E.; A.S.; and A.E.E.; resources, S.E.; A.E.; A.S.; and A.E.E.; data curation, S.E.; A.E.; A.S.; and A.E.E.; writing—original draft preparation, S.E.; N.G.A.; H.G.; M.S.A.; and M.M.; writing—review and editing, S.E.; A.E.; M.S.A.; A.S.; and A.E.E.; visualization, M.S.A.; M.M.; and A.E.; supervision, S.E.; A.E.; and A.S.; project administration, S.E.; H.G.; and A.E.E.; funding acquisition, A.S. All authors have read and agreed to the published version of the manuscript.

Funding: This research received no external funding.

Institutional Review Board Statement: Not applicable.

Informed Consent Statement: Not applicable.

Data Availability Statement: All data are presented within the article.

Conflicts of Interest: The authors declare no conflict of interest.

References

1. Liu B, Xin Q, Zhang M, Chen J, Lu Q, Zhou X, et al. Research progress on mango post-harvest ripening physiology and the regulatory technologies. Foods. 2022;12: 173.
2. L. Alexander DG. Ethylene biosynthesis and action in tomato: a model for climacteric fruit ripening. . J Exp Bot. 2002;53: 2039–2055.
3. Van de Poel B, Vandendriessche T, Hertog ML, Nicolai BM, Geeraerd A. Detached ripening of non-climacteric strawberry impairs aroma profile and fruit quality. Post-

harvest Biol Technol. 2014;95: 70–80.
4. Groff PM, Takahashi SE, Padilha JB, Bochio V, Schadeck MM, Maier GS, et al. Importance of temperature and humidity and the effects of light during incubation offer tile eggs of chickens. Redvet. 2017;18.
5. Saad A, Azam MM, Amer BMA. Quality analysis prediction and discriminating strawberry maturity with a hand-held Vis–NIR spectrometer. Food Anal Methods. 2022; 1–11.
6. Marques EJN, de Freitas ST, Pimentel MF, Pasquini C. Rapid and non-destructive determination of quality parameters in the 'Tommy Atkins' mango using a novel handheld near infrared spectrometer. Food Chem. 2016;197: 1207–1214.
7. Wendel A, Underwood J, Walsh K. Maturity estimation of mangoes using hyperspectral imaging from a ground based mobile platform. Comput Electron Agric. 2018;155: 298–313.
8. Li B-J, Grierson D, Shi Y, Chen K-S. Roles of abscisic acid in regulating ripening and quality of strawberry, a model non-climacteric fruit. Hortic Res. 2022;9: uhac089.
9. Włodarska K, Szulc J, Khmelinskii I, Sikorska E. Non-destructive determination of strawberry fruit and juice quality parameters using ultraviolet, visible, and near-infrared spectroscopy. J Sci Food Agric. 2019;99: 5953–5961.
10. Weng S, Yu S, Dong R, Pan F, Liang D. Nondestructive detection of storage time of strawberries using visible/near-infrared hyperspectral imaging. Int J Food Prop. 2020;23: 269–281.
11. Mishra P, Woltering E. Semi-supervised robust models for predicting dry matter in mango fruit with near-infrared spectroscopy. Postharvest Biol Technol. 2023;200: 112335.
12. Gao Z, Shao Y, Xuan G, Wang Y, Liu Y, Han X. Real-time hyperspectral imaging for the in-field estimation of strawberry ripeness with deep learning. Artif Intell Agric. 2020;4: 31–38.
13. Ping F, Yang J, Zhou X, Su Y, Ju Y, Fang Y, et al. Quality Assessment and Ripeness Prediction of Table Grapes Using Visible–Near-Infrared Spectroscopy. Foods. 2023;12: 2364.
14. Sirisomboon P. NIR spectroscopy for quality evaluation of fruits and vegetables. Mater Today Proc. 2018;5: 22481–22486.
15. Basak JK, Madhavi BGK, Paudel B, Kim NE, Kim HT. Prediction of total soluble solids and pH of strawberry fruits using RGB, HSV and HSL colour spaces and machine learning models. Foods. 2022;11: 2086.
16. Gomes V, Mendes-Ferreira A, Melo-Pinto P. Application of hyperspectral imaging and deep learning for robust prediction of sugar and pH levels in wine grape berries. Sensors. 2021;21: 3459.
17. Fatchurrahman D, Nosrati M, Amodio ML, Chaudhry MMA, de Chiara MLV, Mastrandrea L, et al. Comparison performance of visible-nir and near-infrared hyperspectral imaging for prediction of nutritional quality of goji berry (Lycium barbarum L.). Foods. 2021;10: 1676.
18. Li M, Lv W, Zhao R, Guo H, Liu J, Han D. Non-destructive assessment of quality parameters in 'Friar'plums during low temperature storage using visible/near infrared spectroscopy. Food Control. 2017;73: 1334–1341.
19. Rutkowski KP, Michalczuk B, Konopacki P. Nondestructive determination of 'Golden Delicious' apple quality and harvest maturity. J Fruit Ornam Plant Res. 2008;16: 39–52.
20. Raun WR, Solie JB, Johnson G V, Stone ML, Lukina E V, Thomason WE, et al. In-season prediction of potential grain yield in winter wheat using canopy reflectance. Agron J. 2001;93: 131–138.
21. Zude M, Herold B, Roger J-M, Bellon-Maurel V, Landahl S. Non-destructive tests on

the prediction of apple fruit flesh firmness and soluble solids content on tree and in shelf life. J Food Eng. 2006;77: 254–260.
22. Slaughter DC, Crisosto CH, Tiwari G. Nondestructive determination of flesh color in clingstone peaches. J Food Eng. 2013;116: 920–925.
23. Huang Y, Lu R, Chen K. Assessment of tomato soluble solids content and pH by spatially-resolved and conventional Vis/NIR spectroscopy. J Food Eng. 2018;236: 19–28.
24. Abbaspour-Gilandeh Y, Sabzi S, Hernández-Hernández M, Hernández-Hernández JL, Azadshahraki F. Nondestructive estimation of the chlorophyll b of apple fruit by color and spectral features using different methods of hybrid artificial neural network. Agronomy. 2019;9: 735.
25. Elsayed S, Galal H, Allam A, Schmidhalter U. Passive reflectance sensing and digital image analysis for assessing quality parameters of mango fruits. Sci Hortic (Amsterdam). 2016;212: 136–147.
26. Jiang H, Zhang C, Liu F, Zhu HY, He Y. Identification of strawberry ripeness based on multispectral indexes extracted from hyperspectral images. Guang pu xue yu Guang pu fen xi= Guang pu. 2016;36: 1423–1427.
27. Galal H, Elsayed S, Elsherbiny O, Allam A, Farouk M. Using RGB imaging, optimized three-band spectral indices, and a decision tree model to assess Orange fruit quality. Agriculture. 2022;12: 1558.
28. Galal H, Elsayed S, Allam A, Farouk M. Indirect quantitative analysis of biochemical parameters in banana using spectral reflectance indices combined with machine learning modeling. Horticulturae. 2022;8: 438.
29. Scala K Di, Meschino G, Vega-Galvez A, Lemus-Mondaca R, Roura S, Mascheroni R. An artificial neural network model for prediction of quality characteristics of apples during convective dehydration. Food Sci Technol. 2013;33: 411–416.
30. Torkashvand AM, Ahmadi A, Nikravesh NL. Prediction of kiwifruit firmness using fruit mineral nutrient concentration by artificial neural network (ANN) and multiple linear regressions (MLR). J Integr Agric. 2017;16: 1634–1644.
31. Du C-J, Sun D-W. Recent developments in the applications of image processing techniques for food quality evaluation. Trends food Sci Technol. 2004;15: 230–249.
32. Tripathi MK, Maktedar DD. Internal quality assessment of mango fruit: an automated grading system with ensemble classifier. Imaging Sci J. 2022;70: 253–272.
33. Rad MRN, Fanaei HR, Rad MRP. Application of Artificial Neural Networks to predict the final fruit weight and random forest to select important variables in native population of melon (Cucumis melo L.). Sci Hortic (Amsterdam). 2015;181: 108–112.
34. Huang X, Wang H, Qu S, Luo W, Gao Z. Using artificial neural network in predicting the key fruit quality of loquat. Food Sci Nutr. 2021;9: 1780–1791.
35. Lan H, Wang Z, Niu H, Zhang H, Zhang Y, Tang Y, et al. A nondestructive testing method for soluble solid content in Korla fragrant pears based on electrical properties and artificial neural network. Food Sci Nutr. 2020;8: 5172–5181.
36. Devassy BM, George S. Estimation of strawberry firmness using hyperspectral imaging: a comparison of regression models. J Spectr Imaging. 2021;10.
37. Ribeiro MN, Carvalho IA, Fonseca GA, Lago RC, Rocha LCR, Ferreira DD, et al. Quality control of fresh strawberries by a random forest model. J Sci Food Agric. 2021;101: 4514–4522.
38. Acharya SK, Mishra DP. Current Advances in Mechanical Engineering. 2020.
39. Nagy A, Riczu P, Tamás J. Spectral evaluation of apple fruit ripening and pigment content alteration. Sci Hortic (Amsterdam). 2016;201: 256–264.
40. Rouse Jr JW, Haas RH, Deering DW, Schell JA, Harlan JC. Monitoring the vernal advancement and retrogradation (green wave effect) of natural vegetation. 1974.
41. Sims DA, Gamon JA. Relationships between leaf pigment content and spectral reflec-

tance across a wide range of species, leaf structures and developmental stages. Remote Sens Environ. 2002;81: 337–354.
42. Hossain R, Timmer D. Machine learning model optimization with hyper parameter tuning approach. Glob J Comput Sci Technol D Neural Artif Intell. 2021;21: 31.
43. Oymak S. Stochastic gradient descent learns state equations with nonlinear activations. conference on Learning Theory. PMLR; 2019. pp. 2551–2579.
44. Mijwel MM. Artificial neural networks advantages and disadvantages. Mesopotamian J Big Data. 2021;2021: 29–31.
45. Sharma S, Sharma S, Athaiya A. Activation functions in neural networks. Towar Data Sci. 2017;6: 310–316.
46. Breiman L. Random forests. Mach Learn. 2001;45: 5–32.
47. Han J, Pei J, Tong H. Data mining: concepts and techniques. Morgan kaufmann; 2022.
48. Xia Y, Liu C, Li Y, Liu N. A boosted decision tree approach using Bayesian hyper-parameter optimization for credit scoring. Expert Syst Appl. 2017;78: 225–241.
49. Malone BP, Styc Q, Minasny B, McBratney AB. Digital soil mapping of soil carbon at the farm scale: A spatial downscaling approach in consideration of measured and uncertain data. Geoderma. 2017;290: 91–99.
50. Saggi MK, Jain S. Reference evapotranspiration estimation and modeling of the Punjab Northern India using deep learning. Comput Electron Agric. 2019;156: 387–398.
51. Elsayed S, El-Gozayer K, Allam A, Schmidhalter U. Passive reflectance sensing using regression and multivariate analysis to estimate biochemical parameters of different fruits kinds. Sci Hortic (Amsterdam). 2019;243: 21–33.
52. Medlicott AP, Bhogal M, Reynolds SB. Changes in peel pigmentation during ripening of mango fruit (Mangifera indica var. Tommy Atkins). Ann Appl Biol. 1986;109: 651–656.
53. Lizada C. Mango. Biochem fruit ripening. 1993; 255–271.
54. Palafox-Carlos H, Yahia E, Islas-Osuna MA, Gutierrez-Martinez P, Robles-Sánchez M, González-Aguilar GA. Effect of ripeness stage of mango fruit (Mangifera indica L., cv. Ataulfo) on physiological parameters and antioxidant activity. Sci Hortic (Amsterdam). 2012;135: 7–13.
55. Danquah JA, Roberts CO, Appiah M. Effects of decline in fish landings on the livelihoods of coastal communities in Central Region of Ghana. Coast Manag. 2021;49: 617–635.
56. Subedi PP, Walsh KB, Owens G. Prediction of mango eating quality at harvest using short-wave near infrared spectrometry. Postharvest Biol Technol. 2007;43: 326–334.
57. Islam MS, Aziz MG, Mortuza MG, Rahman MA. Profiling of physical and chemical properties of mango at different ripening stages. J Environ Sci Nat Resour. 2016;9: 121–126.
58. Nambi VE, Thangavel K, Jesudas DM. Scientific classification of ripening period and development of colour grade chart for Indian mangoes (Mangifera indica L.) using multivariate cluster analysis. Sci Hortic (Amsterdam). 2015;193: 90–98.
59. Gonzalez-Aguilar GA, Celis J, Sotelo-Mundo RR, De La Rosa LA, Rodrigo-Garcia J, Alvarez-Parrilla E. Physiological and biochemical changes of different fresh-cut mango cultivars stored at 5 C. Int J Food Sci Technol. 2008;43: 91–101.
60. Robles-Sánchez RM, Islas-Osuna MA, Astiazarán-García H, Vazquez-Ortiz FA, Martín-Belloso O, Gorinstein S, et al. Quality index, consumer acceptability, bioactive compounds, and antioxidant activity of fresh-cut "Ataulfo" mangoes (Mangifera indica L.) as affected by low-temperature storage. J Food Sci. 2009;74: S126–S134.
61. Winarno FG. Kimia Pangan Dan Gizi Gramedia. Jakarta; 1997.
62. Cahyono B. Sukses Budidaya Stroberi di Pot dan Perkebunan. Penerbit Andi Jogyakarta. 2011.

63. Janurianti NMD, Utama IMS, Gunam IBW. Colour and quality of strawberry fruit (Fragaria x ananassa Duch.) at different levels of maturity. SEAS (Sustainable Environ Agric Sci. 2021;5: 22–28.
64. Tsormpatsidis E, Ordidge M, Henbest RGC, Wagstaffe A, Battey NH, Hadley P. Harvesting fruit of equivalent chronological age and fruit position shows individual effects of UV radiation on aspects of the strawberry ripening process. Environ Exp Bot. 2011;74: 178–185.
65. Chang J-C, Chang M-W. 'Elongated Fruit No. 1'Mulberry: An Elite Cultivar for Fresh Consumption. J Am Pomol Soc. 2010;64: 101.
66. Salamat R, Ghassemzadeh HR, Heris SSS, Hajilou J. Determination of appropriate harvesting time for strawberry to enhance its flavor index and reduce bruising susceptibility. 2013.
67. Ullah I. Development, characterization and evaluation of watermelon, mango, pears and lime juice blend. 1990.
68. Bhatti MS. Studies on some ripening changes in mangoes during storage. M Sc(Hons) Thesis Dep Food Technol Univ Agric Faisalabad. 1975.
69. Martínez GA, Chaves AR, Civello PM. β-Xylosidase activity and expression of a β-xylosidase gene during strawberry fruit ripening. Plant Physiol Biochem. 2004;42: 89–96.
70. Rahman MM, Moniruzzaman M, Ahmad MR, Sarker BC, Alam MK. Maturity stages affect the postharvest quality and shelf-life of fruits of strawberry genotypes growing in subtropical regions. J Saudi Soc Agric Sci. 2016;15: 28–37.
71. Gunness P, Kravchuk O, Nottingham SM, D'Arcy BR, Gidley MJ. Sensory analysis of individual strawberry fruit and comparison with instrumental analysis. Postharvest Biol Technol. 2009;52: 164–172.
72. de Jesús Ornelas-Paz J, Yahia EM, Ramírez-Bustamante N, Pérez-Martínez JD, del Pilar Escalante-Minakata M, Ibarra-Junquera V, et al. Physical attributes and chemical composition of organic strawberry fruit (Fragaria x ananassa Duch, Cv. Albion) at six stages of ripening. Food Chem. 2013;138: 372–381.
73. Liu C, Liu W, Lu X, Ma F, Chen W, Yang J, et al. Application of multispectral imaging to determine quality attributes and ripeness stage in strawberry fruit. PLoS One. 2014;9: e87818.
74. Mancini M, Mazzoni L, Gagliardi F, Balducci F, Duca D, Toscano G, et al. Application of the non-destructive NIR technique for the evaluation of strawberry fruits quality parameters. Foods. 2020;9: 441.
75. Weng S, Yu S, Guo B, Tang P, Liang D. Non-destructive detection of strawberry quality using multi-features of hyperspectral imaging and multivariate methods. Sensors. 2020;20: 3074.
76. Tijskens LMM, Zerbini PE, Schouten RE, Vanoli M, Jacob S, Grassi M, et al. Assessing harvest maturity in nectarines. Postharvest Biol Technol. 2007;45: 204–213.
77. Wei K, Ma C, Sun K, Liu Q, Zhao N, Sun Y, et al. Relationship between optical properties and soluble sugar contents of apple flesh during storage. Postharvest Biol Technol. 2020;159: 111021.
78. Yahaya OKM, Jafri MZM, Aziz AA, Omar AF. Simplified optical fiber RGB system in evaluating intrinsic quality of Sala mango. Opt Eng. 2015;54: 67108.
79. Mithun BS, Mondal M, Vishwakarma H, Shinde S, Kimbahune S. Detection of artificially ripened mango using spectrometric analysis. Sensing for Agriculture and Food Quality and Safety IX. SPIE; 2017. pp. 68–76.
80. Munawar AA, Suhandy D. Fast and contactless assessment of intact mango fruit quality attributes using near infrared spectroscopy (NIRS). IOP Conference Series: Earth and Environmental Science. IOP Publishing; 2021. p. 12028.

81. Zhang Z, Wang T, Fan H. Neural Network-Based Analysis and Its Application to Spectroscopy for Mango. Appl Sci. 2024;14: 2402.
82. Amoriello T, Ciorba R, Ruggiero G, Amoriello M, Ciccoritti R. A Performance Evaluation of Two Hyperspectral Imaging Systems for the Prediction of Strawberries' Pomological Traits. Sensors. 2023;24: 174.
83. ElMasry GM, ElSheikh IH, Morsy NE. Colour grading of strawberry using computer vision and backpropagation artificial neural network. J Soil Sci Agric Eng. 2009;34: 7063–7077.

Figure
Click here to access/download;Figure;Figures.docx

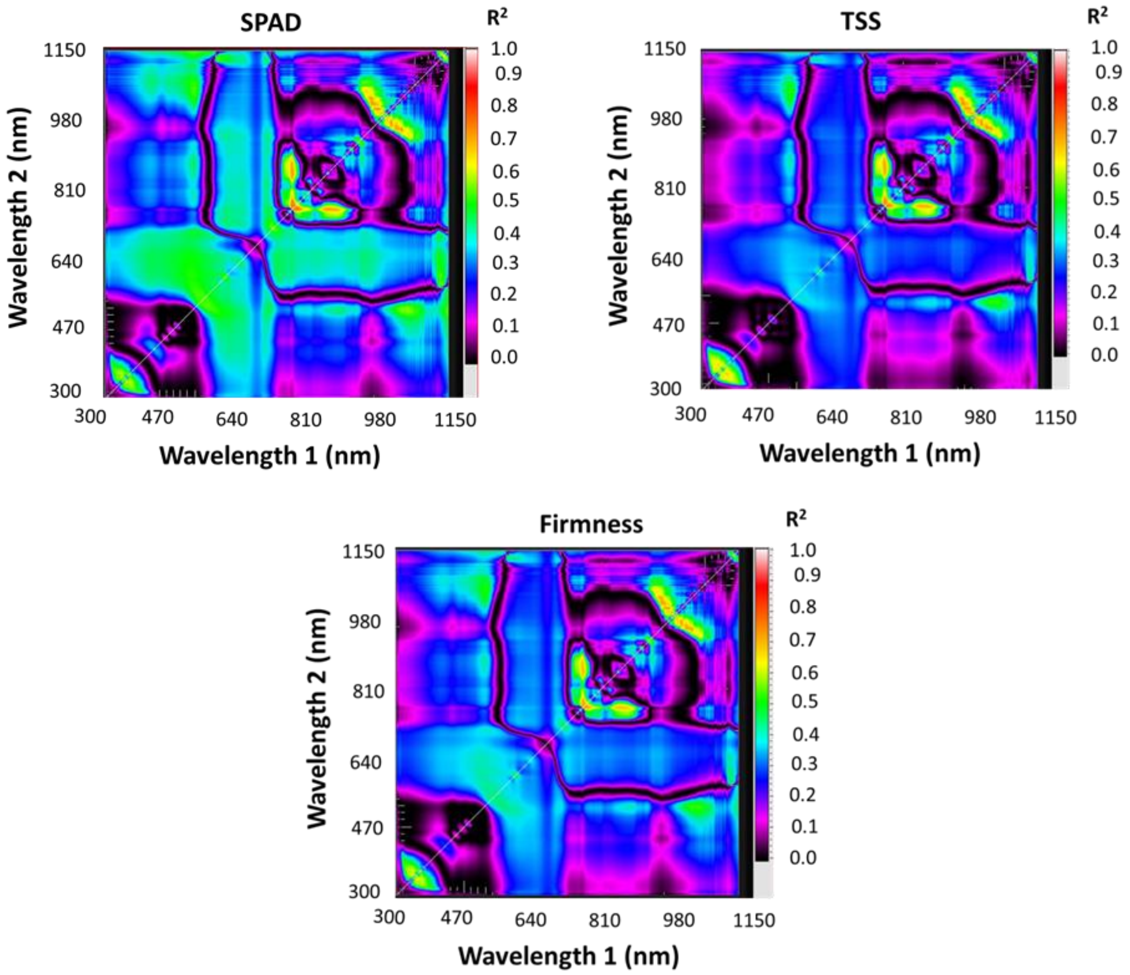

Figure 1. Correlation matrices displaying (R^2) values for possible dull wavelength together ranging from 302 to 1148 nm with SPAD, total soluble solids (TSS), and firmness of mango fruits.

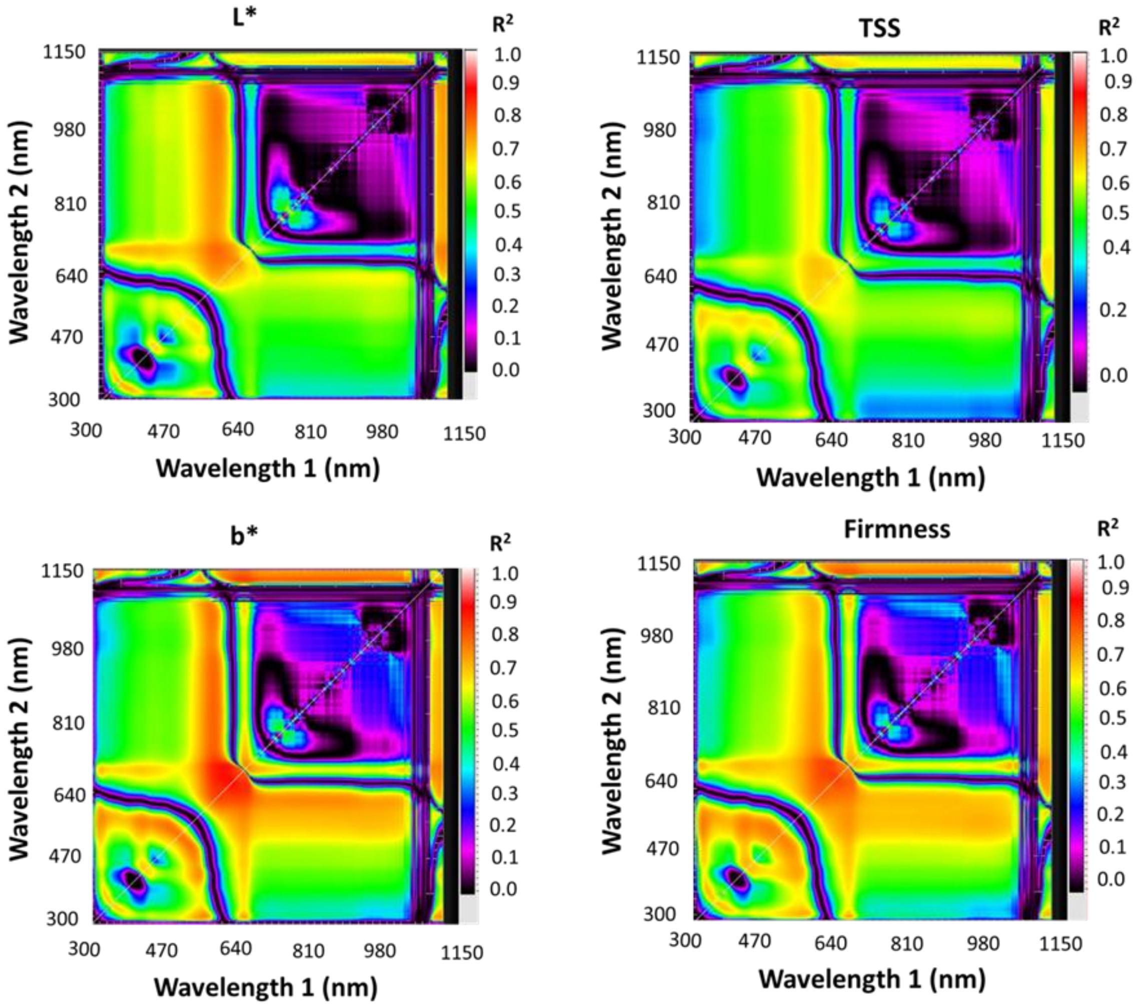

Figure 2. Correlation matrices displaying (R^2) values for possible dull wavelength together ranging from 302 to 1148 nm with total L*, b*, total soluble solids (TSS), and firmness of strawberry fruits.

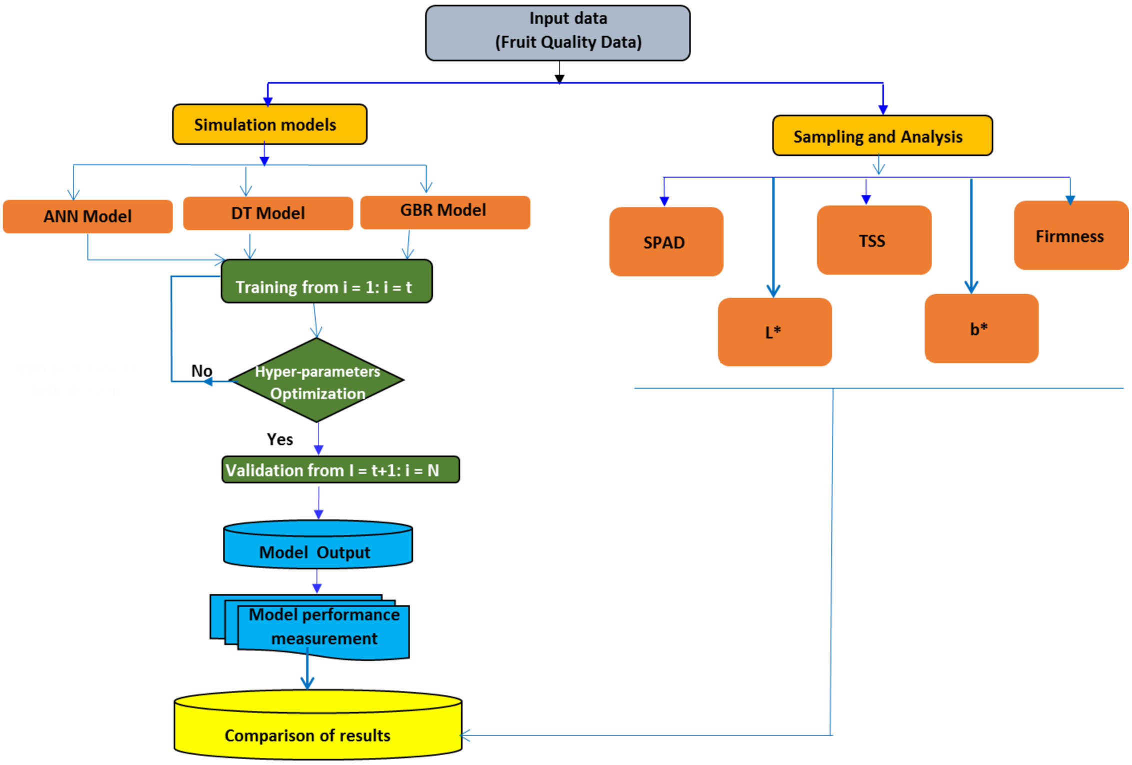

Figure 3. Schematic diagram of the methodology presented in this study.

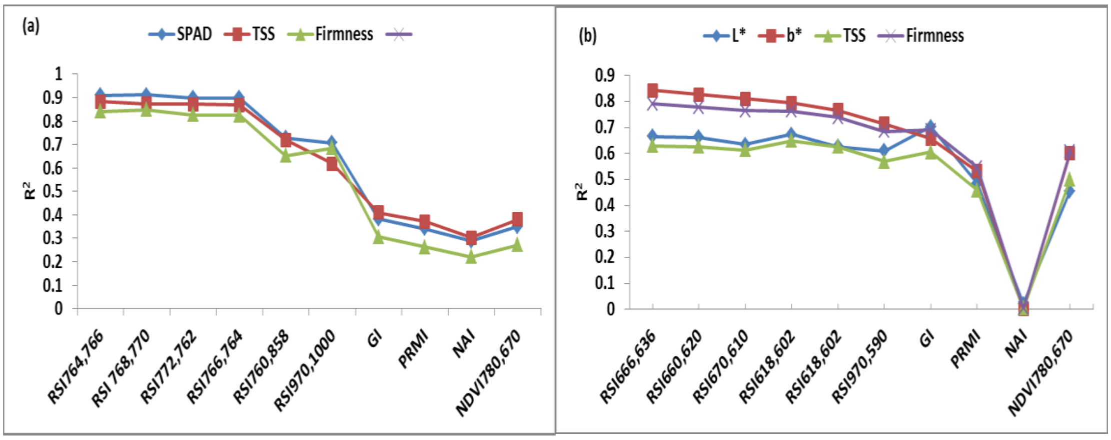

Figure 4. Coefficient of determination values of linear regression models of (a) three mango fruit attributes as SPAD, total soluble solids (TSS), and firmness with ten spectral reflectance indices (SRIs) as well as (b) four strawberry fruit attributes as L*, b*, TSS and firmness with ten spectral reflectance indices.

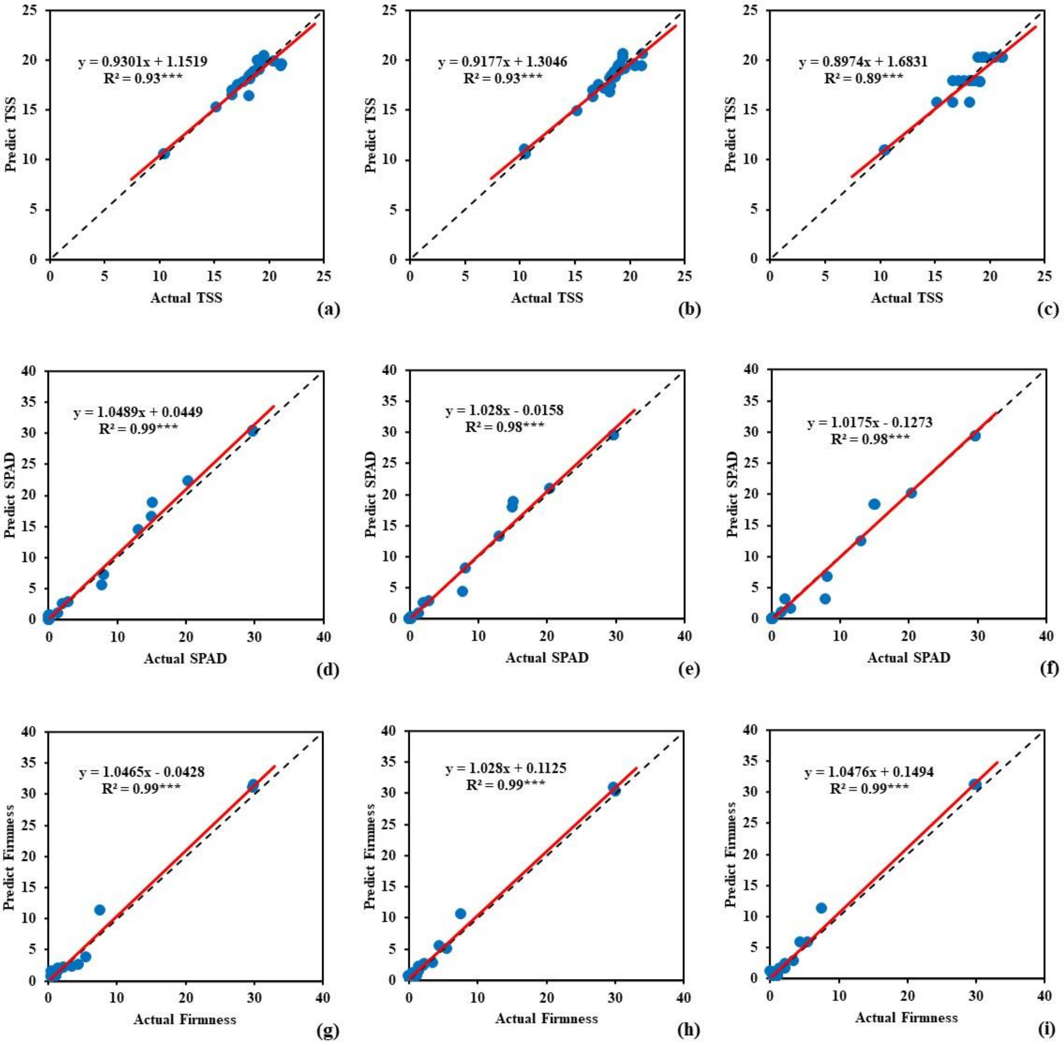

Figure 5. Comparative Analysis of (a, d, g) artificial neural network, (b, e, h) random forest, and (c, f, i) decision trees for TSS, SPAD, and firmness for mango during testing phase.

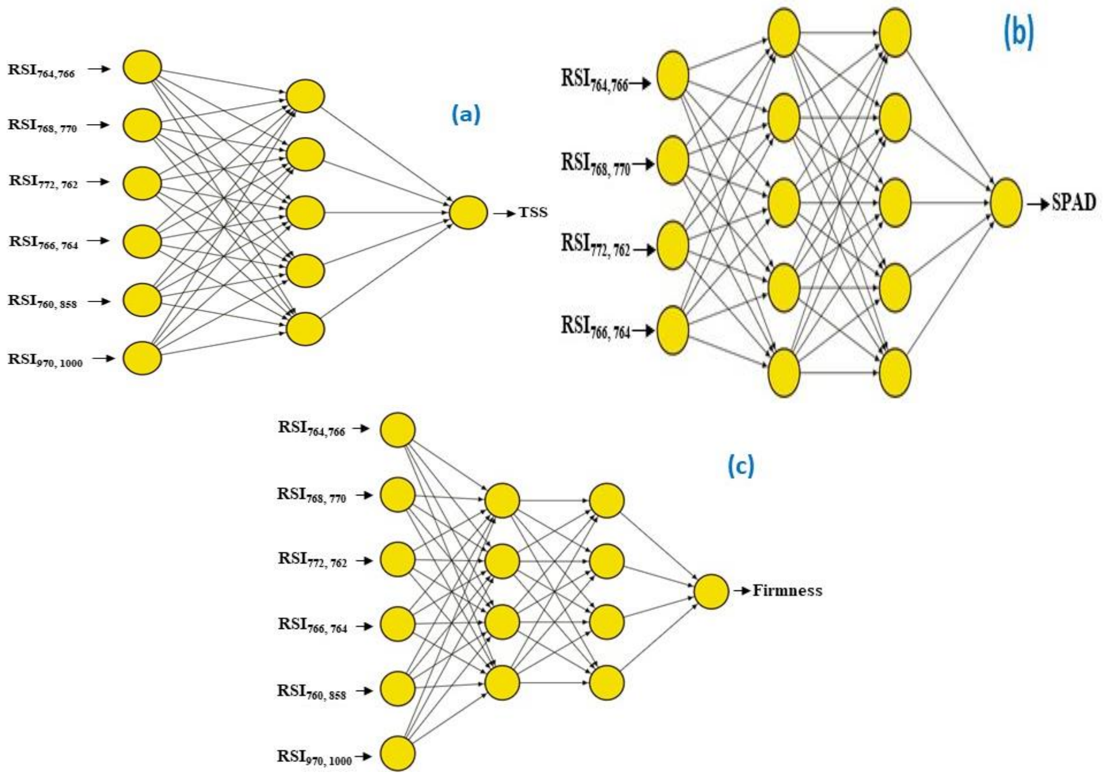

Figure 6. ANN architecture, incorporating a combination of the effective variables, for identifying TSS, SPAD, and firmness.

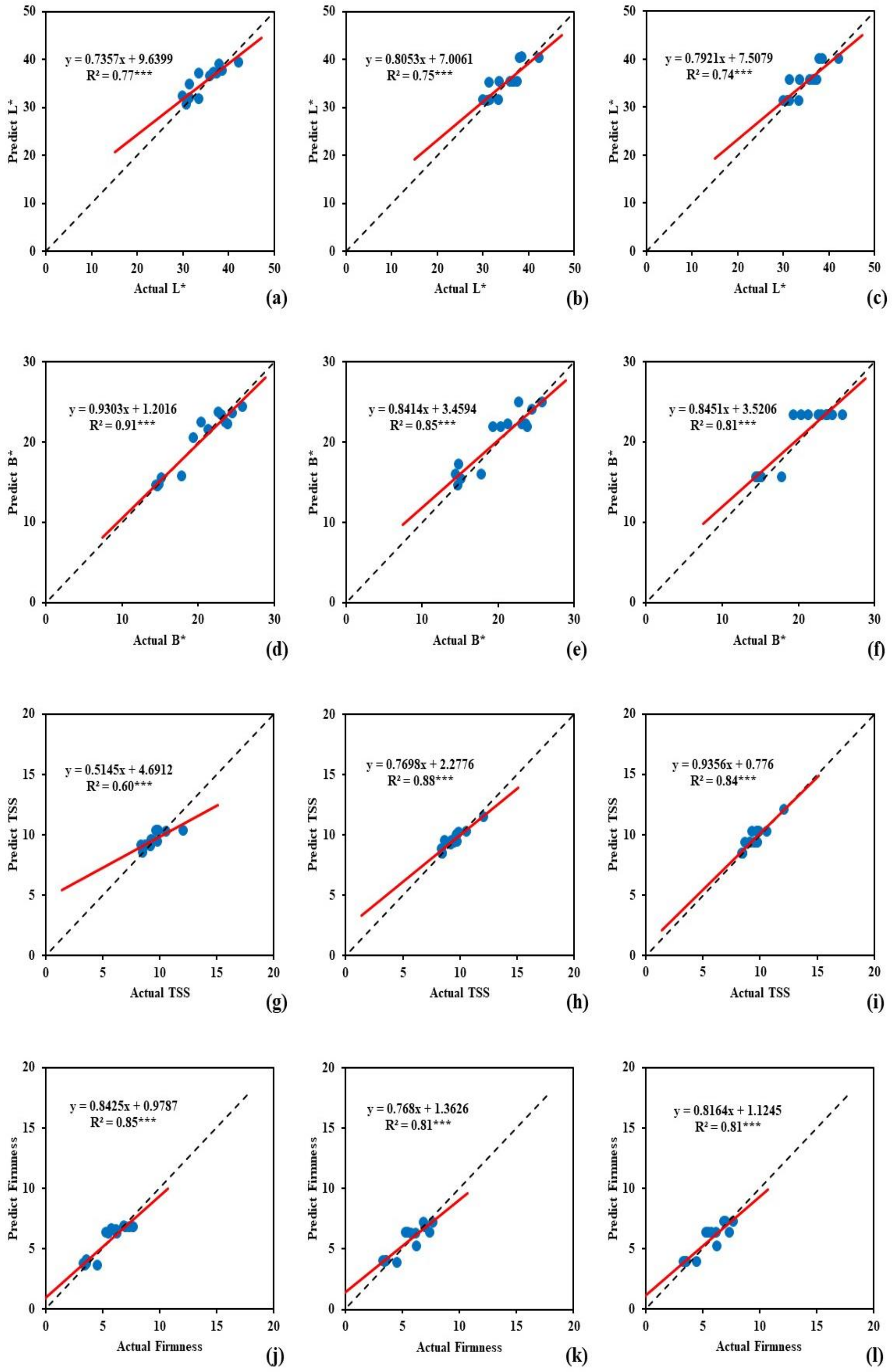

Figure 7. Comparative Analysis of (a, d, g, j) Artificial Neural Network, (b, e, h, k) Random Forest, and (c, f, i, l) Decision Trees for L*, b*, TSS, and firmness for strawberry during testing phase.

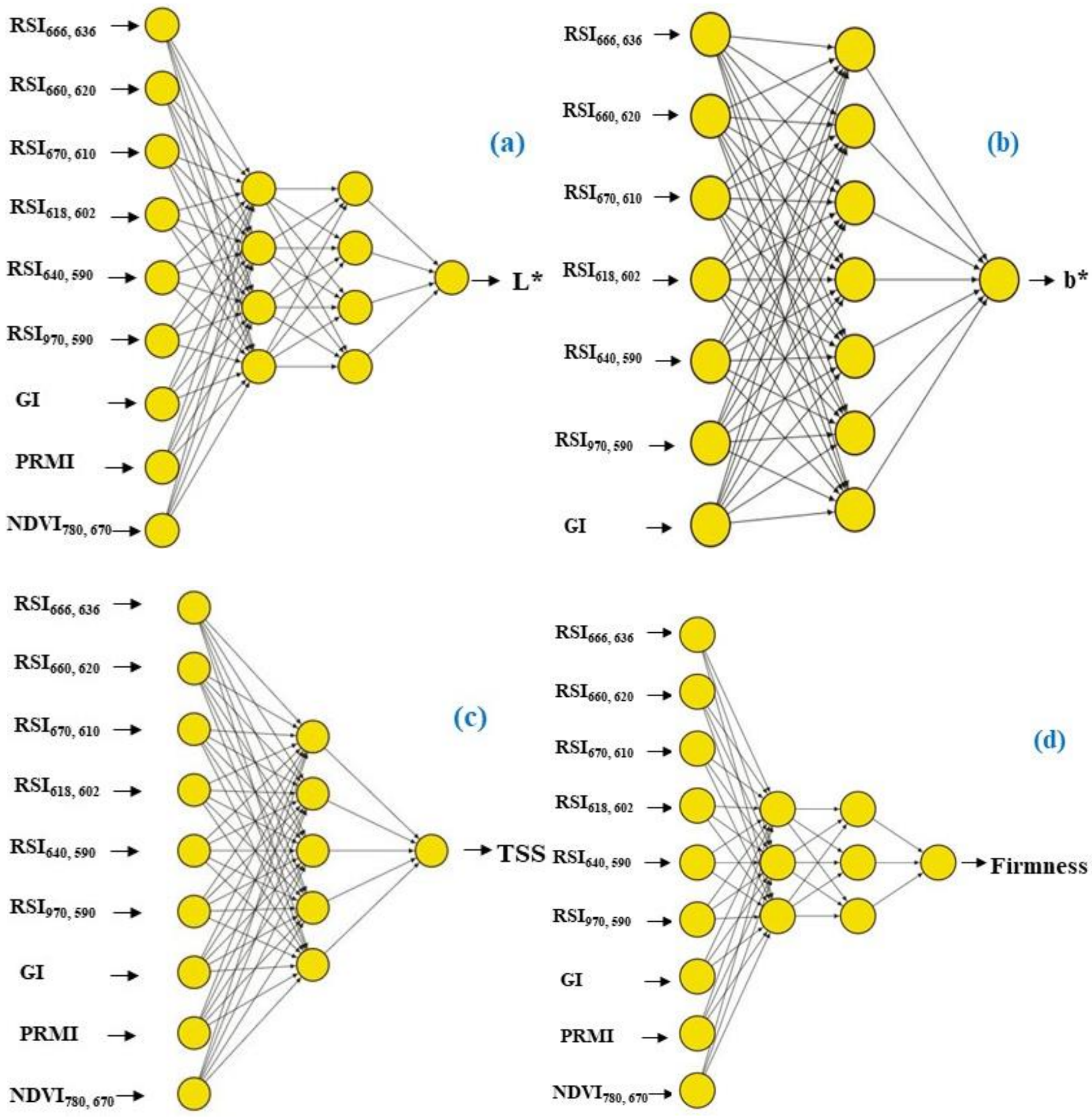

Figure 8. ANN architecture, incorporating a combination of the effective variables, for identifying L, b, TSS, and firmness.
