# Supplementary material for: Hyperspectral technology and machine learning models to estimate the fruit quality parameters of mango and strawberry crops
Source: PLoS One. 2025 Feb 11;20(2):e0313397. doi: 10.1371/journal.pone.0313397 (PMC11813159; doi:10.1371/journal.pone.0313397)
Supplement: Attachment — Submitted filename: Response_editors.docx [file pone.0313397.s002.docx]

| Q1 | In the online submission form, you indicated that "Data will be available upon request"  All PLOS journals now require all data underlying the findings described in their manuscript to be freely available to other researchers, either  1. In a public repository,  2. Within the manuscript itself, or  3. Uploaded as supplementary information.  This policy applies to all data except where public deposition would breach compliance with the protocol approved by your research ethics board. If your data cannot be made publicly available for ethical or legal reasons (e.g., public availability would compromise patient privacy), please explain your reasons on resubmission and your exemption request will be escalated for approval. |
| --- | --- |
| A1 | Thank you. It I modified in the revised version of the manuscript. All data, Code, raw data of Mango and strawberry are presented in the Manuscript and supporting information files |
| Q2 | Can you please upload an additional copy of your revised manuscript that does not contain any tracked changes or highlighting as your main article file. This will be used in the production process if your manuscript is accepted. Please amend the file type for the file showing your changes to Revised Manuscript w/tracked changes. Please follow this link for more information: http://blogs.PLOS.org/everyone/2011/05/10/how-to-submit-your-revised-manuscript/ |
| A2 | The clean revised version is uploaded |
| Q3 | In your Methods section, please provide additional information regarding the permits you obtained for the work. Please ensure you have included the full name of the authority that approved the field site access and, if no permits were required, a brief statement explaining why. |
| A3 | Thank you for your comment  This research was conducted at some farms in Sadat city on mango and strawberry fruits to carry out the analyses required. There is no permission for this research because of non-including animals or humans in the work. |
